# Comparative effectiveness and safety of Chinese medicine belly button application for childhood diarrhea: a Bayesian network meta-analysis of randomized controlled trials

**DOI:** 10.3389/fped.2023.1180694

**Published:** 2023-08-04

**Authors:** Zhi-jun Bu, Yan-ni Liu, Md. Shahjalal, You-you Zheng, Cheng-jiang Liu, Meng-meng Ye, Jin-yang Xu, Xin-yao Peng, Xue-hui Wang, Xu Chen, Jian-ping Liu, Hui-lan Liu, Zhao-lan Liu

**Affiliations:** ^1^ Centre for Evidence-Based Chinese Medicine, Beijing University of Chinese Medicine, Beijing, China; ^2^ Beijing University of Chinese Medicine, Third Affiliated Hospital, Beijing University of Chinese Medicine, Beijing, China; ^3^ Department of Public Health, North South University, Dhaka, Bangladesh; ^4^ Department of General Medicine, Affiliated an Qin First People’s Hospital of Anhui Medical University, Anqin, China; ^5^ School of Laboratory Medicine, Hubei University of Chinese Medicine, Wuhan, China

**Keywords:** Chinese medicine, belly button application, childhood diarrhea, network meta-analysis, NMA

## Abstract

**Background:**

Chinese medicine belly button application (CMBBA) has been used to treat childhood diarrhea (CD) in several randomized controlled trials (RCTs), but its effectiveness and combination strategy still need to be clarified. Therefore, we aimed to evaluate the effectiveness, safety, and the optimal combination strategy of CMBBA in treating CD.

**Methods:**

Up until January 2023, we searched for studies that met our inclusion criteria in six databases, including PubMed, the Cochrane Library, Chinese SinoMed, CNKI, VIP, and Wanfang. Heterogeneity was quantified using *I*^2^ statistics. A methodological evaluation was performed using the Cochrane Risk Bias Tool 2.0. The Confidence in Network Meta-Analysis online software was employed to evaluate evidence grading. A minimally contextualized framework was used to provide a comprehensive conclusion for the network meta-analysis. This study protocol was registered with PROSPERO.

**Results:**

We analyzed data from 33 RCTs that included 4,490 children with diarrhea. In terms of clinical effectiveness, CMBBA plus montmorillonite powder plus anti-infectives may be the most effective treatment option for children with diarrhea and concurrent infection according to a minimally contextualized framework. Either exclusive use of CMBBA or CMBBA in combination with modern medicine was beneficial in reducing the time to diarrhea disappearance (MD = −1.33 days, 95% CI: −1.59 to −1.08, *Z* = −10.103, *p* < 0.001) compared to modern medicine exclusively, and the difference was statistically significant. The combined usage of CMBBA could shorten the recovery time of dehydration by an average of 0.74 days (MD = −0.74 days, 95% CI: −1.10 to −0.37, *Z* = −3.931.103, *p* < 0.001). While some studies have reported mild allergic reactions and mild abdominal pain after CMBBA use, these symptoms can be cured in a relatively short period of time.

**Conclusions:**

The combination of CMBBA, montmorillonite powder, and anti-infectives may provide superior clinical effectiveness for children with diarrhea and concurrent infection. To treat CD, CMBBA can be used effectively and safely. However, the findings must be interpreted with cautiously due to the limited number of clinical trials and the low quality of the studies. In addition, the choice of treatment plan should also be based on the specific conditions of each patient.

**Systematic Review Registration:**

https://www.crd.york.ac.uk/prospero/, identifier: CRD42022380694

## 1. Introduction

Childhood diarrhea (CD) is a common childhood digestive tract disease characterized by increased stool frequency and changes in stool characteristics caused by multiple pathogens and factors. It is also one of the most common diseases in infants and young children in China (1). In addition to abnormal stool, children with diarrhea are also accompanied by additional symptoms, such as fever, abdominal pain, and abdominal distension; in severe cases, there are varying degrees of dehydration, electrolyte imbalance, systemic infection poisoning symptoms, and even death (2). The common causes of diarrhea in children are viral and bacterial infections. For bacterial infections, Shigella is the most common pathogen spectrum in poor areas, while *Escherichia coli*, Salmonella, and Yersinia are the most common in economically developed areas. As for viral infections, rotavirus is the leading cause of diarrhea (3, 4). According to the World Health Organization (WHO), diarrhea is the second leading cause of death among children under five, with an annual incidence of 1.7 billion and 525,000 deaths worldwide. In China, the incidence of diarrheal diseases in the whole population is 0.17–0.70 times per person-year, and the incidence in children under five is 2.50–3.38 times per person-year (5).

At present, the routine treatment of CD mainly includes fluid replacement to relieve the child's dehydration state, microecologics to regulate the intestinal flora environment, intestinal mucosal protectors to enhance the intestinal mucosal barrier, zinc to improve the prognosis, and treatment for different pathogens, as well as symptomatic treatment for fever and abdominal pain (6). In the above treatment, however, fluid rehydration only alleviates dehydration in children and does not treat CD. The prophylaxis of the mucosal lining of the gut is prolonged, and the oral medication is bitter, making it difficult for children to take. It's well documented that antibiotic treatments are prone to causing bacterial disorders (7).

Chinese medicine belly button application (CMBBA) is a traditional Chinese medicine local treatment method that grinds Chinese medicine into powder, mixes it with vinegar to form a paste, and applies it to the navel area. The skin absorbs the active ingredients of CMBBA, which can then produce clinical therapeutic effects. Commonly used Chinese medicines for CMBBA include Cinnamomum verum *[Lauraceae]*, Syzygium aromaticum *[Myrtaceae]*, and Evodia rutaecarpa *[Rutaceae]* (8). Pharmacological studies have shown that Evodia rutaecarpa can inhibit the tension and amplitude of gastrointestinal contractions, suppress abdominal movements, and relieve intestinal spasms (9). Syzygium aromaticum contains various active ingredients such as eugenol, butyl p-hydroxybenzoate, thymol, and caryophyllene, which have effects such as slowing down gastrointestinal emptying, anti-gastric ulcer, antiemetic, analgesic, anti-inflammatory, and antibacterial (10). Cinnamomum verum’s volatile oil and its component cinnamaldehyde can relax guinea pig ileum longitudinal muscle *in vitro*, inhibit mouse gastrointestinal propulsion, and have a significant antagonistic effect on mouse diarrhea induced by castor oil and senna leaves (11). The use of vinegar in the production of CMBBA can increase the solubility of Chinese medicine, which is beneficial for its penetration, absorption, and effectiveness.

It’s well documented that CMBBA has unique advantages in the treatment of CD. Firstly, the navel is located in the middle of the childhood abdominal navel with a rich network of capillaries. Through skin absorption stimulation, CMBBA directly acts on the lesion site, and can quickly exert the pharmacological effects of the drug (12). Secondly, compared with bitter tasting oral medications, external administration using CMBBA is easier for children to accept. In addition, CMBBA is convenient, painless, non-invasive, and avoids damage to the gastrointestinal, liver, and kidney functions caused by drugs, reduces the first-pass effect of the liver, significantly improves the local lesion site drug concentration, and prolongs the duration of drug effectiveness (13). Therefore, CMBBA has broad application prospects in the treatment of CD. Although we have made significant progress in improving children's health and well-being in recent years, we can still make more. According to the WHO report in 2020, two regions, Sub-Saharan Africa and Central and Southern Asia, that account for 52% of the global population of under five children and also accounted for more than 80% of deaths caused by CD and acute respiratory diseases such as pneumonia (14). This report clearly shows that CD is one of the major risk factors for endangering children's health. Indeed, Sustainable Development Goals (SDG), adopted by all United Nations Member States in 2015, pledge to “leave no one behind”. Target 3 emphasizes ensuring healthy lives, promoting well-being for all ages, and reducing premature death (15). To meet SDGs-target, it is necessary for us to find a variety of effective treatment measures and the most effective combination strategy of treatment measures for CD. As one of the important treatment measures of Chinese medicine, CMBBA has a unique effect on the treatment of CD. To date, to our knowledge, a growing number of RCTs about CMBBA for the treatment of CD have appeared, but systematic review for its effectiveness, safety, and optimal combination strategy has not yet been systematically demonstrated. As a result, we conducted a Network Meta-Analysis (NMA) to assess the effectiveness, safety, and optimal combination strategy of CMBBA in treating of CD. The authors expect that the findings from this study can inform clinicians of a new approach to treating CD using CMBBA in a wide range, resulting in less pain and happier growth for patients with CD.

## 2. Materials and methods

To begin with, we submitted our application to PROSPERO under registration CRD4202380694. Then, we carried out this NMA based on the guidelines provided by the Preferred Reporting Items for Systematic Review (PRISMA) 2020 (16) and the Cochrane Handbook for Systematic Reviews of Interventions (17). We provided the PRISMA checklist in Supplementary File S1.

### 2.1. Eligibility criteria

(1) Study type: RCTs.
(2) Study subjects: The patient was under 18 years old and was clearly diagnosed with diarrheal disease, regardless of nationality, age, sex, ethnicity, or course of illness.
(3) Interventions: The intervention group used CMBBA or CMBBA combined with other modern medicine.
(4) Outcomes: Primary outcomes included clinical effectiveness [according to the criteria for the effectiveness of Traditional Chinese Medicine disease diagnosis and treatment and the standard of Chinese diarrhea disease diagnosis and treatment plan (1)]: Healing: the stool traits and frequency returned to normal, and the clinical symptoms disappeared. Effectiveness: The stools’ characteristics and frequency were reduced, and the clinical symptoms improved. Ineffectiveness: Neither the trait nor the frequency of stools improved. Total effectiveness = [(healing + effectiveness)/total number of cases]. Secondary outcomes included the recovery time of dehydration, time to diarrheal disappearance, and adverse events.

### 2.2. Search strategy

We searched PubMed, the Cochrane Library, Chinese SinoMed, CNKI, VIP, and Wanfang to select peer-reviewed papers for our NMA. Our search included articles published in Chinese and English language between 1999 and 2020. To retrieve information, a combination of Medical Subject Headings (MeSH) and free-text words was used. The search strategies for each database are presented in Supplementary File S2.

### 2.3. Study selection

The authors Z-JB and Y-NL read the titles, abstracts, and full texts independently to filter the retrieved studies. Relevant information from the studies was extracted, such as the basic information on the study and the outcome data. After the independent screening, the third author, Y-YZ, examined the relevant information extracted from the Z-JB and Y-NL studies, and if the extracted information from the respective studies of Z-JB and Y-NL was found to be inconsistent, the three discussed it in a group to resolve the issue. The data extraction criteria included: first author, time of publication, sample size (number of people in the intervention group, number of people in the control group), age, course of disease, treatment in the intervention group, treatment in the control group, duration of treatment, number of clinically effective people in the intervention group, number of clinically effective people in the control group, adverse effects, and other outcome measures.

### 2.4. Risk of bias

The authors JY-X and X-YP conducted their own assessments of the risk of bias in the included studies using the Cochrane Bias Risk Assessment 2.0 tool (17), and any disagreements were negotiated with X-C and ultimately agreed upon. We assessed the quality of the included studies based on the following criteria: (a) random sequence generation; (b) allocation concealment; (c) blinding of participants and personnel; (d) blinding of outcome assessment; (e) incomplete outcome data; (f) selective reporting; (g) other bias. Each factor was rated as follows: (a) low risk; (b) some concerns; and (c) high risk.

### 2.5. Data analysis

The statistical analysis was performed using gemtc package in the R statistical software (version 3.6.1). Relative risk (RR) and 95% confidence intervals (CI) were used as effect size indicators for dichotomous variables. The mean difference (MD) and 95% CI were considered as effect size measures for continuous variables. The number of pre-iterations and the number of iterations is set to 20,000 and 50,000, respectively. Trajectory plots, density plots, and Brooks-Gelman-Rubin diagnostic plots were used to determine whether a satisfactory degree of convergence has been achieved. If the trial showed good homogeneity in study design, participants, interventions, controls, and outcomes, a random-effects model would be used for this study's analysis. If there was heterogeneity between the results of the study (*I*^2^ > 50% or *p* < 0.1), further analysis of the source of heterogeneity was needed. Sensitivity analysis, subgroup analysis, or meta-regression can be used to explore the sources of heterogeneity. We used the node-splitting method to perform the inconsistency test. For outcome measures that did not form a network, we used direct comparisons. Funnel plots were used to assess publication bias in relevant studies.

### 2.6. Quality of evidence assessment

According to the Confidence in Network Meta-Analysis (CINeMA) evaluation manuals, Y-NL and M-MY evaluated the included studies from six aspects: within-study bias, reporting bias, indirectness, imprecision, heterogeneity, and incoherence (18). The CINeMA evaluation website is https://cinema.ispm.unibe.ch/.

### 2.7. A minimally contextualised framework

We employed the minimally contextualized framework to interpret our results. The framework is based on two fundamental principles: interventions should be categorized by their effectiveness or harmfulness, ranked from most to least effective/harmful, and judgments assigning interventions to categories should consider effect estimates, evidence certainty, and rankings simultaneously (19). This methodology facilitates an objective and scientifically sound interpretation and presentation of research results while minimizing the influence of subjective value judgments, thereby enhancing the findings' reliability and credibility.

## 3. Results

### 3.1. Study selection

**Figure 1:**
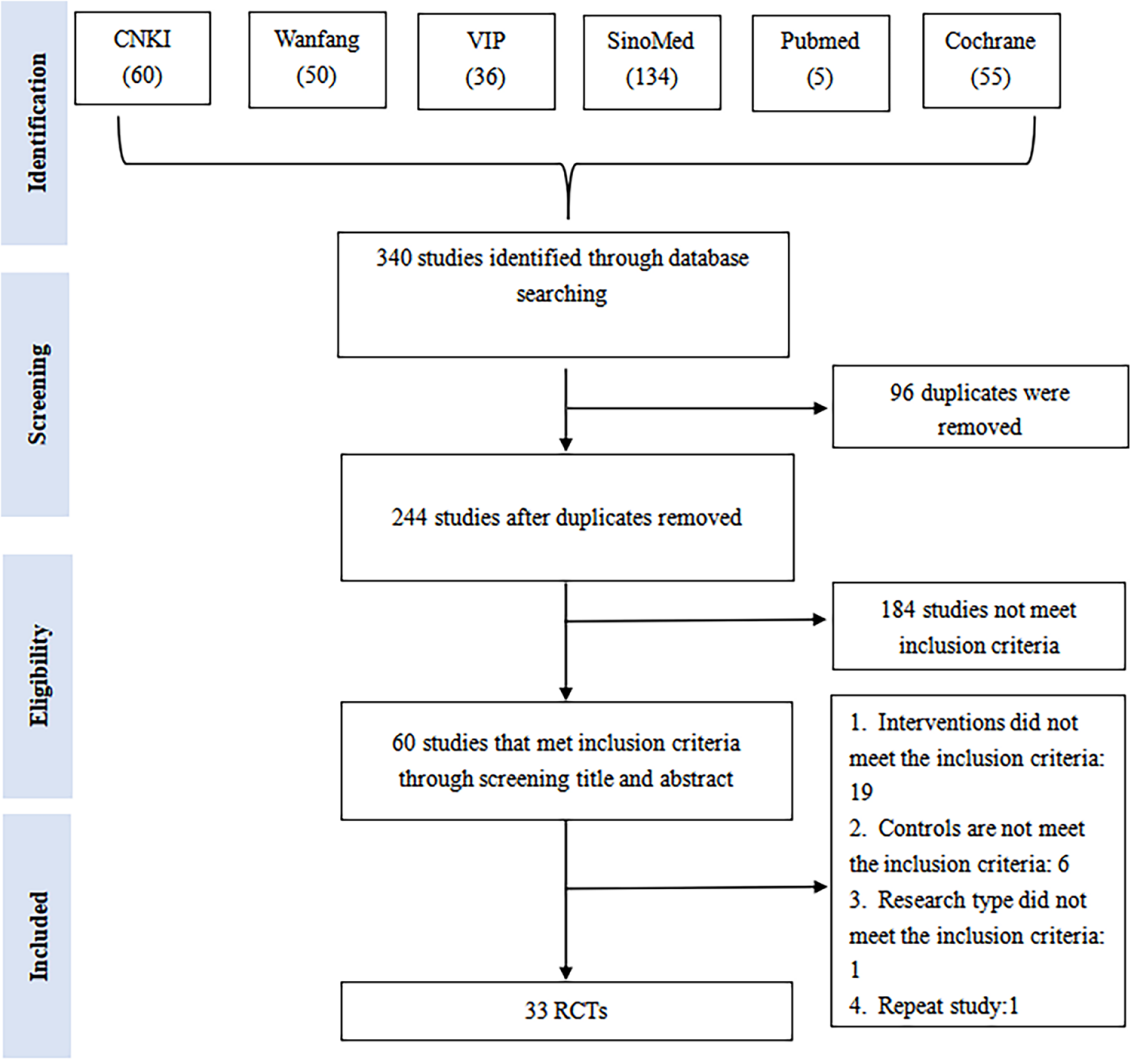
A flowchart of study screening and selection. CNKI, China national knowledge infrastructure; SinoMed, the Chinese biomedical literature database; WanFang, the WanFang database; VIP, the Chinese scientific journals full-text database.

We retrieved 340 studies from six databases. Upon re-examination, 96 studies were found to be duplicates using EndNote (EN) X9.3.3, and after a preliminary screening of the remaining 244 studies, 184 were found not to meet the inclusion criteria. After a full-text screening of the remaining 60 studies, we found 33 RCTs that could be included in our NMA. A flowchart of study screening and selection can be seen in Figure 1.

### 3.2. Study characteristics

In total, 33 studies published between 1999 and 2020 were included in our NMA. This included 4,490, children with diarrhea: 2,319 in the control group and 2,171 in the intervention group. Of these, 3 studies compared CMBBA to montmorillonite powder plus microecologics; 5 studies compared CMBBA to montmorillonite powder; and 1 study compared CMBBA to montmorillonite powder plus anti-infectives. Three studies compared CMBBA with montmorillonite powder plus anti-infectives plus microecologics. One study compared CMBBA plus montmorillonite powder to montmorillonite powder plus microecologics. Nine studies compared CMBBA plus montmorillonite powder to montmorillonite powder. Seven studies compared CMBBA plus montmorillonite powder plus microecologics and with montmorillonite powder plus microecologics. Four studies compared CMBBA plus montmorillonite powder plus anti-infectives and montmorillonite powder plus anti-infectives. Table 1 displays the general characteristics of the included studies.

**Table 1:** The detailed characteristics of the included studies.

Study ID	Gender (male/female)	Sample size (I/C)	Age (mean or range, years)	Course (mean or range, days)	Intervention vs. control	Intervention details	Control details	Outcomes
Chen (20)	67/43	I, 55 C, 55	I, 1.07 ± 0.40 C, 1.04 ± 0.36	I, 14.94 ± 2.43 C, 14.68 ± 1.96	CBMMA + MP vs. MP	CMBBA, 24 h/time, QD	NR	① ④
Qian (21)	31/29	I, 30 C, 30	I, 4.39 ± 1.48 C, 4.31 ± 1.32	I, 4.01 ± 1.29 C, 4.03 ± 1.23	CBMMA + MP vs. MP	CMBBA, 3∼6 h/time, BID, + Control. Course = 3 day	MP, <1 year old, 1/3 pack/time, 1–2 years old, 1/3–2/3 pack/time, >2 years old, 2/3–1 pack/time. TID. Course = 3 day	① ④
Gui (22)	73/63	I, 68 C, 68	I, 1.69 ± 0.33 C, 1.63 ± 0.31	NR	CMBBA vs. MP	CMBBA, QD. Course = 5 day	MP, 1 pack/time, <1 year old, QD, ≥1 year old, BID, Course = 5 day	① ②
Zhou (23)	78/42	I, 60 C, 60	I, 1.31 ± 0.34 C, 1.3 ± 0.43	NR	CBMMA + MP + M vs. MP + M	CMBBA, 2–6 h/time, BID, +Control. Course = 3 day	M, 1 pack/time, BID, MP, 1/3 pack/time, TID, Course = 3 day	① ②
Huang (24)	51/45	I, 48 C, 48	I, 0.58–3 C, 0.5–3	I, 0.29–3 C, 0.25–2	CBMMP + MP vs. MP	CMBBA, 10 h/time, BID, + Control. Course = 3 day	MP, <1 year old, 1/3 pack/time, TID, 1–2 years old, 1/3∼2/3 pack/time, TID, >2 years old, 2/3–1 pack/time, TID. Course = 3 day	① ② ③
Chen (25)	65/55	I, 60 C, 60	I, 1.48 C, 1.52	I, 2.55 C, 2.67	CMBBA + MP vs. MP + M	CMBBA, 3 h/time, QD, Course = 3 day	M, <1 year old, 1/2 pack/time, 1–3 years old, 1 pack/time, MP: NR. TID. Course = 3 day	①
Yue (26)	152/128	I, 140 C, 140	I, 1.19 ± 0.56 C, 1.16 ± 0.58	I, 7.6 ± 1.0 C, 7.4 ± 0.8	CMBBA vs. MP + M	CMBBA, 4–6 h/time, QD, Course = 5 day	NR	①
Huang (27)	46/36	I, 41 C, 41	I, 0.85 ± 0.19 C, 0.9 ± 0.20	<3	CMBBA + MP vs. MP	CMBBA, 12–24 h/time	NR	① ② ③ ④
Ren (28)	55/33	I, 44 C, 44	I, 1 ± 0.38 C, 1 ± 0.08	NR	CMBBA + MP + AI vs. MP + AI	CMBBA, +Control. Course = 2 day	MP, <1 year old, 1 pack/time, >1 year old, 2 pack/time. BID. AI, NR. Course = 2 day	① ④
Tan (29)	27/33	I, 30 C, 30	2.3	<3	CMBBA + MP + M vs. MP + M	CMBBA, 24 h/time, QD, +Control. Course = 3 day	MP, <1 year old, 1/3 pack/time, 1–2 years old, 1/3–2/3 pack/time, >2 years old, 2/3–1 pack/day. TID. M, <1 year old, 1/6 pack/time, 1–5 years old, 1/3 pack/time. TID. Course = 3 day	①
Wu (30)	68/52	I, 60 C, 60	2.5	2–6	CMBBA + M + AI vs. MP + AI	CMBBA, BID, +Control. Course = 3 day	MP, <1 year old, 1/3 pack/time, 1–3 years old, 1/2 pack/time, >3 years old, 1 pack/time. TID. AI, NR. Course = 3 day	①
Xin (31)	NR	I, 80 C, 80	0.08–3	1–30	CBMMP + MP vs. MP	CMBBA, 48 h/time, +Control. Course = 6 day	MP, <1 year old, 1/3 pack/time, 1–3 years old, 1/2 pack/time. The first dose is doubled. TID. Course = 3 day	①
Wang (32)	98/94	I, 96 C, 96	0.5–0.75 (30) 0.83–0.92 (82) 1–2 (80)	NR	CMBBA + MP + AI vs. MP + AI	CMBBA, QD, +Control. Course = 3 day	AI, Ribavirin. Antibiotic. MP: NR. Course = 3 day	①
Dong (33)	NR	I, 96 C, 90	1.8	1.6	CMBBA + MP + M vs. MP + M	CMBBA, 8 h/time, BID, +Control. Course = 3 day	NR	① ② ③ ④
Li (34)	151/103	I, 127 C, 127	0.25–1 (68) 1–3 (152) 4–5 (34)	≤14	CBMMP + MP + M vs. MP + M	CMBBA, QD, 24 h/time, +Control. Course = 3 day	MP, <6 kg, 1/3 pack/time, 6–15 kg, 1/2 pack/time, >15 kg, 1 pack/time. M: NR. TID. Course = 3 day	①
Shi (35)	60/40	I, 50 C, 50	I, 0.3–5 C, 0.2–6	NR	CMBBA + MP + AI vs. MP + AI	CMBBA, QD, +Control. Course = 3 day	AI, Ribavirin, 10 mg/(kg·day). Amoxicillin. MP, 1 pack/time TID. Course = 3 day	① ② ③
Zhang (36)	NR	I, 52 C, 52	NR	<2	CMBBA + MP + M vs. MP + M.	CMBBA, 20 h/time, QD, +Control. Course = 3 day	NR	①
Zhang (37)	NR	I, 30 C, 30	0.5–3	<14	CBMMA vs. MP	CMBBA, QD, 2–4 h/time. Course = 5 day	MP, 0.5–1 year old, 1 pack/time, 1–2 years old, 1.5 pack/time, 2–3 years old, 2 pack/time. TID. Course = 5 day	①
Zhou (38)	37/31	I, 36 C, 32	I, 0.90 C, 0.91	NR	CMBBA vs. MP + M + AI.	CMBBA, 24 h/time, QD, Course = 5 day	AI, Ribavirin	①
Li (39)	74/46	I, 60 C, 60	I, 0–0.5 (6) 0.5–1 (22) 1–2 (32) C, 0–0.5 (9) 0.5–1 (22) 1–2 (29)	I, 0–3 (39) ≥3 (21) C, 0–3 (38) ≥3 (22)	CMBBA vs. MP + AI	CMBBA, QD, Course = 3 day	MP, 1/3–1/2 pack/time, TID. AI, Moroxydine, 5 mg–10 mg/kg/day, TID. Course = 3 day	①
Luo (40)	76/52	I, 64 C, 64	I, 1.01 ± 0.75 C, 1.05 ± 0.82	I, 1–2 (21) 3–4 (36) >5 (7) C, 1–2 (27) 3–4 (36) >5 (7)	CMBBA vs. MP + M.	CMBBA, 24 h/time, QD, Course = 3 day	M, <2 years old, 1 pack/time, >2 years old, 1–2 pack/time. QD or BID. MP, <1 year old, 1/3 pack/time, 1–2 years old, 1/3–2/3 pack/time, >2 years old, 2/3–1 pack/time. TID. Course = 3 day	①
Chen (41)	85/61	I, 76 C, 70	0.25–2	>2	CMBBA + MP + M vs.MP + M	CMBBA, 24 h/time, QD, +Control. Course = 3 day	MP, TID. M, TID. Course = 3 day	①
Yang (42)	46/24	I, 35 C, 35	I, 1.01 ± 0.78 C, 1.04 ± 0.82	I, ≤2 (17) 3–5 (8) 5–10 (7) 11–30 (3) C, ≤2 (19) 3–5 (7) 5–10 (7) 11–30 (2)	CMBBA vs. MP	CMBBA, 6 h/time, QD, Course = 3 day	NR	①
Zhu (43)	37/51	I, 48 C, 40	I, 3.8 ± 0.41 C, 3.92 ± 0.38	I, 1.15–7.00 C, 1.20–6.85	CMBBA + MP vs. MP	CMBBA, QD, +Control, Course = 6 day	MP, TID. Course = 6 day	①
Fu (44)	NR	I, 74 C, 72	0.25–3	<3	CMBBA vs. MP + M.	CMBBA, 12–24 h/time, Course = 3 day	MP, <1 year old, 1/3 pack/time, 1–2 years old, 2/3 pack/time. The first dose is doubled. TID. M, <1 year old, 1/2 pack/time, 1–2 years old, 1 pack/time. BID. Course = 3 day	①
Li (45)	64/52	I, 64 C, 52	I, 1.5 C, 1.42	I, 2.7 C, 2.8	CBMMP + MP vs. MP	CMBBA, QD. + Control. Course = 3–5 day	MP, 0–1 years old, 1/3 pack/time, 1–2 years old, 1/2–2/3 pack/time, 2∼3 years old, 5/6–1 pack/time. The first dose is doubled. TID. Course = 3–5	① ② ④
Wang (46)	110/100	I, 112 C, 98	I, 0–1 (78) >1 (34) C, 0–1 (71) >1 (27)	NR	CMBBA vs. MP	CMBBA, 48 h/time. Course = 3 day	MP, <1 year old, 1 pack/time, 1–2 years old, 1–2 pack/time. QD. Course = 3 day	①
Zheng (47)	128/72	I, 100 C, 100	I, 1.0 C, 1.1	I, 1–2 (35) 3–4 (50) >5 (15) C, 1–2 (38) 3–4 (54) >5 (8)	CMBBA vs. MP + M + AI	CMBBA, 24 h/time, QD, Course = 5 day	AI, Ribavirin. Antibiotic. Course = 5 day	①
Zhou (48)	128/72	I, 100 C, 100	I, 1.01 ± 0.75 C, 1.05 ± 0.82	I, 1–2 (35) 3–4 (50) >5 (15) C, 1–2 (38) 3–4 (54) >5 (8)	CMBBA vs. MP + M + AI	CMBBA, 24 h/time, QD, Course = 5 day	AI, Ribavirin. Course = 5 day	①
Chen (49)	75/55	I, 70 C, 60	I, 0.5–1 (42) 1–2 (28) C, 0.5–1 (35) 1–2 (25)	I, 1–3 C, 1–4	CBMMA + MP + M vs. MP + M	CMBBA, 12–24 h/time, +Control. Course = 3 day	NR	① ②
Zhao (50)	64/52	I, 64 C, 52	I, 1.5 C, 1.42	I, 2.7 C, 2.8	CMBBA + MP vs. MP	CMBBA, QD, + Control, Course = 3–5 day	MP, 0–1 years old, 1/3 pack/time, 1–2 years old, 1/2–2/3 pack/time, 2–3 years old, 5/6–1 pack/time. The first dose is doubled. TID. Course = 3–5 day	① ② ④
Zhou (51)	77/67	I, 96 C, 48	I, 0.79 C, 0.83	NR	CBMMA vs. MP	CMBBA, QD, Course = 3 day	MP, <1 year old, 1 pack/time, 1–3 years old, 1.5 pack/time. TID. Course = 3day	①
Tan (52)	188/92	I, 153 C, 127	I, 0.7 ± 0.23 C, 0.74 ± 0.23	I, 2.1 ± 0.64 C, 2.3 ± 0. 69	CMBBA + MP vs. MP	CMBBA, 24 h/time, QD, +Control. Course = 3 day	NR	①

I, intervention group; C, control group; NM, not mentioned; qd, one time a day; bid, two times a day; tid, three times a day. The specific meaning of treatment column are, CMBBA, Chinese medicine belly button application; MP, montmorillonite powder; M, microecologics; AI, anti-infectives. ① Clinical effectiveness, ② time to diarrheal disappearance, ③ the recovery time of dehydration, ④ adverse events. Montmorillonite powder every pack, 3 g. Microecologics every pack, 1 g.

### 3.3. Risk of bias of included studies

**Figure 2:**
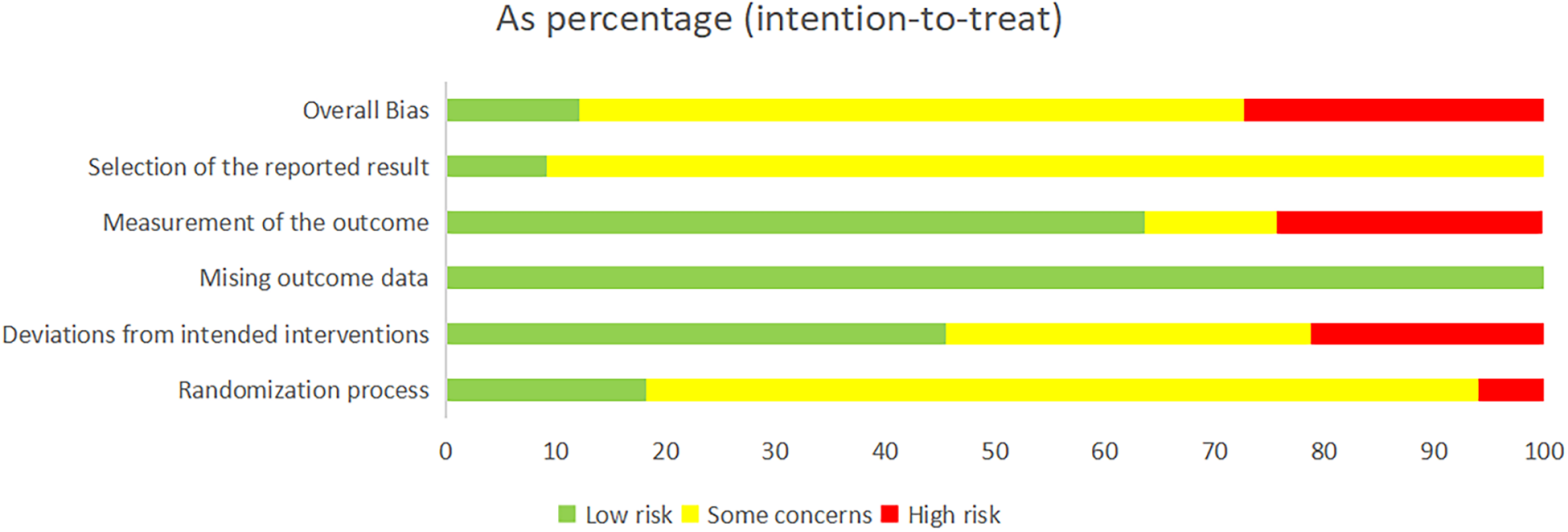
The risk of bias charts for the included studies.

For random sequence generation, 33 studies mentioned the use of randomization schemes, but only 3 studies used random number table methods, 1 study used sealed envelope methods, 1 study used piecewise randomization methods, and 2 studies were randomly assigned according to the order of admission. The other studies did not specify a specific randomization method. In terms of baseline balance, all 33 studies had baseline balance. Since this study belongs to the field of Chinese medicine extrinsic therapy, it may be difficult to establish a blind approach in practice, so there was a general bias toward a blind approach in the 33 studies. Regarding the bias of missing outcome data, all studies included established outcome data. In terms of selective reporting bias, it was difficult to determine whether there was a possibility of selective reporting due to some studies published years from the current longer. In total, 4 studies were low risk, 9 studies were high risk, and 20 studies were of some concern. The risk of bias charts for the included studies are shown in Figure 2.

### 3.4. Clinical effectiveness

#### 3.4.1. Pairwise meta-analysis

We performed a pairwise meta-analysis of clinical effectiveness in Supplementary File S3. Pairwise meta-analysis results showed that after combined use of CMBBA or use CMBBA exclusively, the intervention group of clinical effectiveness was higher than the control group (RR: 1.16, 95% CI: 1.13–1.19, *I*^2^ = 31%, *p* = 0.046). Among all the treatments compared, the use of CMBBA exclusively and montmorillonite powder plus microecologics plus anti-infectives was the most obvious clinical effectiveness comparison (RR: 1.26, 95% CI: 1.16–1.38, *I*^2^ = 0%, *p* = 0.940). A subgroup analysis was performed for those with slightly higher heterogeneity among treatment measures. Between the exclusive use of CMBBA and the use of montmorillonite powder (RR: 1.13, 95% CI: 1.05–1.21, *I*^2^ = 42%, *p* = 0.125), we performed a subgroup analysis using the treatment course and found that the heterogeneity was significantly reduced after subgroup analysis. Between CMBBA plus montmorillonite powder plus microecologics and montmorillonite powder plus microecologics (RR: 1.14, 95% CI: 1.08–1.21, *I*^2^ = 43%, *p* = 0.104), we also performed a subgroup analysis of the dehydration and found that the heterogeneity was also significantly reduced after subgroup analysis. Thus, we considered that treatment course and dehydration may be two of the main sources of heterogeneity in clinical effectiveness. The results of the subgroup analysis are presented in Supplementary File S4. We performed sensitivity analyses on all studies and discovered that the results were robust and reliable (*p* < 0.05). The forest plot for sensitivity analysis can be seen in Supplementary File S5.

#### 3.4.2. Network meta-analysis

**Figure 3:**
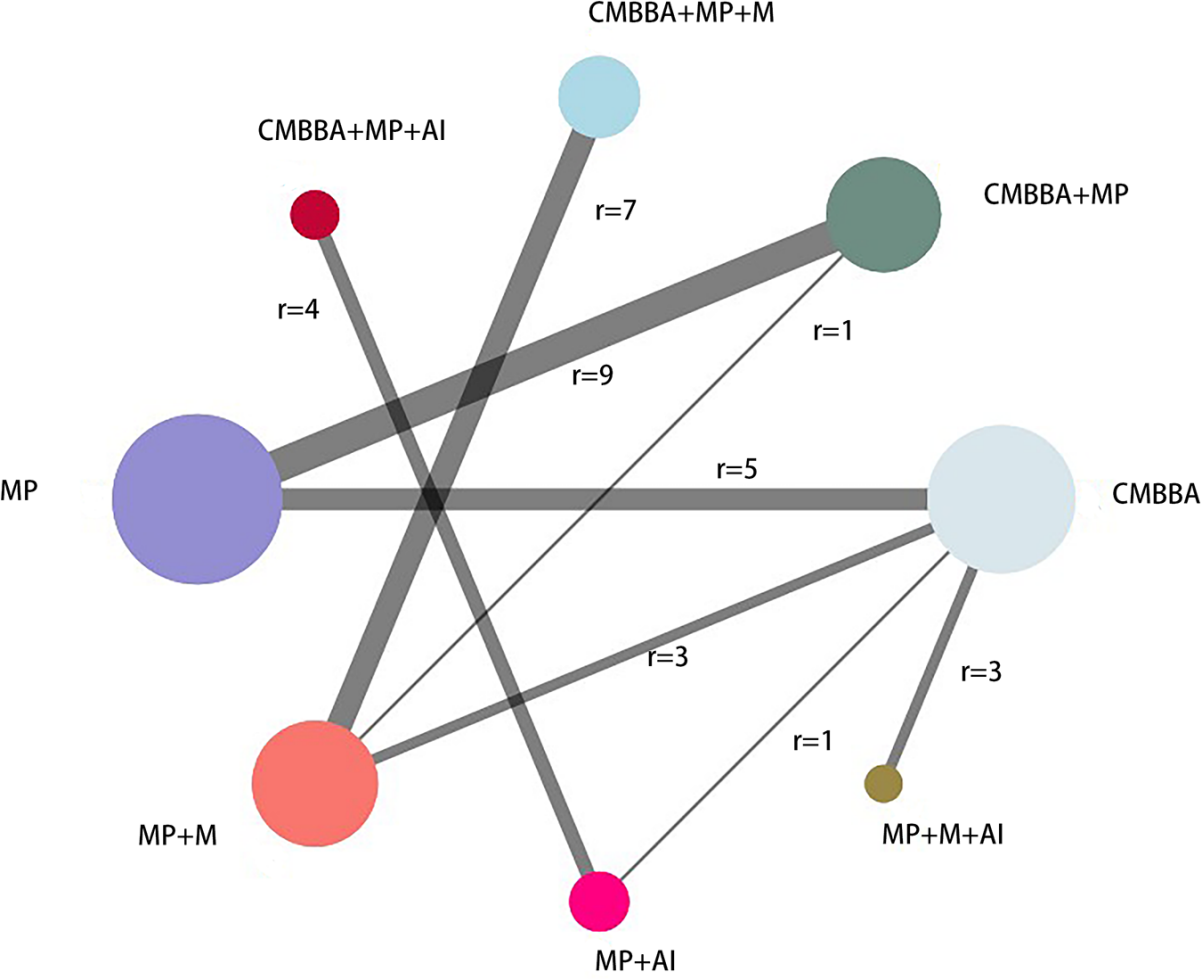
The network diagram of the clinical effectiveness. CMBBA, Chinese medicine belly button application; MP, montmorillonite powder; M, microecologics; AI, anti-infectives. r: The number of studies for the comparison. The node represents an intervention, and each edge represents a head-to-head comparison between two different interventions. The sizes of nodes and edges display the numbers of patients receiving the treatment.

Figure 3 depicts the network diagram of the clinical effectiveness of CMBBA in the treatment of CD. We presented the trajectory plots, density plots, and Brooks-Gelman-Rubin diagnostic plots in Supplementary Files S6, S7, from which we can find that the model converges well. In terms of heterogeneity, we discovered that it was all *I*^2^ < 50% in both the NMA and pairwise meta-analysis. The heterogeneity analysis results of NMA can be seen in Supplementary File S8.

Table 2 shows the comparative effects of RRs and corresponding 95% Cis for different interventions in detail. From Table 2, we can see that the exclusive or combined use of CMBBA was more effective than the exclusive use of modern medicine. And most of the treatments were statistically significant. Statistically significant treatments were shown in bold and underlined in Table 2. From Table 2, we can find that compared with montmorillonite powder plus anti-infectives plus microecologics, CMBBA exclusively (RR = 1.26, 95% CI: 1.15–1.4), CMBBA plus montmorillonite powder (RR = 1.32, 95% CI: 1.17–1.51), CMBBA plus montmorillonite powder plus microecologics (RR = 1.26, 95% CI: 1.1–1.45), CMBBA plus montmorillonite powder plus anti-infectives (RR = 1.36, 95% CI: 1.11–1.65), and montmorillonite powder exclusively (RR = 1.13, 95% CI: 1.01–1.27) can improve the clinical effectiveness. Compared with montmorillonite powder plus microecologics, CMBBA exclusively (RR = 1.14, 95% CI: 1.06–1.23), CMBBA plus montmorillonite powder (RR = 1.2, 95% CI: 1.08–1.33), CMBBA plus montmorillonite powder plus microecologics (RR = 1.13, 95% CI: 1.08–1.21), and CMBBA plus montmorillonite powder plus anti-infectives (RR = 1.23, 95% CI: 1.01–1.48) can improve the clinical effectiveness. Compared with montmorillonite powder plus anti-infectives, CMBBA plus montmorillonite powder plus anti-infectives (RR = 1.21, 95% CI: 1.11–1.32) can improve the clinical effectiveness. Compared with montmorillonite powder exclusively, CMBBA exclusively (RR = 1.11, 95% CI: 1.05–1.19) and CMBBA plus montmorillonite powder (RR = 1.17, 95% CI: 1.11–1.24) can improve the clinical effectiveness. All the above comparisons were statistically significant. In addition, we used the CINeMA evaluation results to label each treatment measure in Table 2. We found that the evidence grading of the CINeMA evaluation between statistically significant treatment measures was also better.

**Table 2:** Network meta-analysis comparisons for clinical effectiveness.

CMBBA	1.05^c^ (0.97, 1.14)	1^c^ (0.91, 1.1)	1.08^c^ (0.9, 1.28)	**0.9**^a^ (**0.84, 0.95)**	**0.88**^a^ (**0.81, 0.95)**	0.9^c^ (0.76, 1.03)	**0.79**^b^ (**0.72, 0.87)**
0.95^c^ (0.88, 1.03)	CMBBA + MP	0.95^c^ (0.84, 1.07)	1.03^c^ (0.84, 1.24)	**0.86**^a^ (**0.8, 0.9)**	**0.84**^a^ (**0.75, 0.93)**	0.86^c^ (0.71, 1)	**0.76**^b^ (**0.66, 0.86)**
1^c^ (0.91, 1.1)	1.05^c^ (0.93, 1.18)	CMBBA + MP + M	1.09^c^ (0.88, 1.31)	0.9^c^ (0.8, 1)	**0.88**^a^ (**0.83, 0.93)**	0.9^c^ (0.74, 1.06)	**0.8**^a^ (**0.69, 0.91)**
0.92^c^ (0.78, 1.11)	0.97^c^ (0.81, 1.19)	0.92^c^ (0.76, 1.13)	CMBBA + MP + AI	0.83^a^ (0.69, 1.01)	**0.81**^a^ (**0.68, 0.99)**	**0.83**^a^ (**0.76, 0.9)**	**0.73**^b^ (**0.61, 0.91)**
**1.11**^a^ (**1.05, 1.19)**	**1.17**^a^ (**1.11, 1.24)**	1.11^c^ (1, 1.25)	1.2^a^ (0.99, 1.45)	MP	0.98^c^ (0.89, 1.08)	1^c^ (0.84, 1.17)	**0.88**^c^ (**0.78, 0.99)**
**1.14**^a^ (**1.06, 1.23)**	**1.2**^a^ (**1.08, 1.33)**	**1.13**^a^ (**1.08, 1.21)**	**1.23**^a^ (**1.01, 1.48)**	1.02^c^ (0.93, 1.12)	MP + M	1.02^c^ (0.85, 1.2)	0.9^c^ (0.8, 1.02)
1.11^c^ (0.97, 1.31)	1.17^c^ (1, 1.41)	1.11^c^ (0.94, 1.35)	**1.21**^a^ (**1.11, 1.32)**	1^c^ (0.86, 1.19)	0.98^c^ (0.83, 1.18)	MP + AI	0.88^c^ (0.75, 1.07)
**1.26**^b^ (**1.15, 1.4)**	**1.32**^b^ (**1.17, 1.51)**	**1.26**^a^ (**1.1, 1.45)**	**1.36**^b^ (**1.11, 1.65)**	**1.13**^c^ (**1.01, 1.27)**	1.11^c^ (0.98, 1.26)	1.13^c^ (0.94, 1.34)	MP + M + AI

CMBBA, Chinese medicine belly button application; MP, montmorillonite powder; M, microecologics; AI, anti-infectives. Statistically significant results were in bold and underscored. The certainty of the evidence (according to Supplementary CINeMA results) was incorporated in this table.

Bold values indicate that the difference is statistically significant.

^a^ Moderate quality of evidence.

^b^ Low quality of evidence.

^c^ Very low quality of evidence.

**Figure 4:**
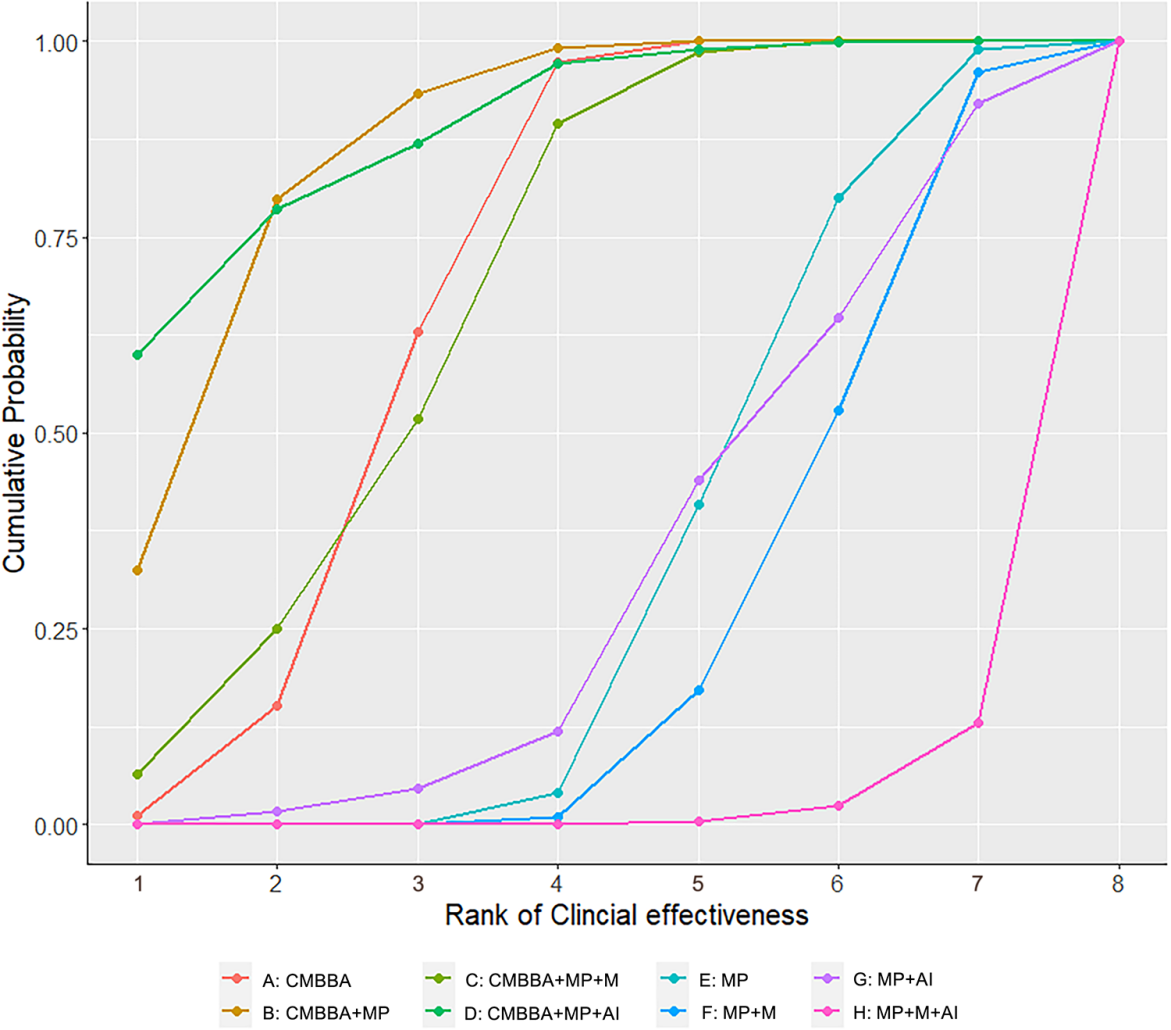
Cumulative probability of ranking clinical effectiveness treatment measures. The higher the ranking, the higher the clinical effectiveness. CMBBA, Chinese medicine belly button application; MP, montmorillonite powder; M, microecologics; AI, anti-infectives.

The numerical results of SUCRA (surface under the cumulative ranking curve) showed that CMBBA plus montmorillonite powder plus anti-infectives may be the best choice for the treatment of CD, while montmorillonite powder plus anti-infectives plus microecologics for the treatment of CD were not outstanding. The SUCRA values of specific treatment measures were ranked as follows: CMBBA plus montmorillonite powder plus anti-infectives (SUCRA: 88%) >CMBBA plus montmorillonite powder (SUCRA: 86%) >CMBBA exclusively (SUCRA: 68%) = CMBBA plus montmorillonite powder plus microecologics (SUCRA: 68%) >montmorillonite powder exclusively (SUCRA: 32%) >montmorillonite powder plus anti-infectives (SUCRA: 31%) >montmorillonite powder plus microecologics (SUCRA: 24%) >montmorillonite powder plus anti-infectives plus microecologics (SUCRA: 0.02%). In addition, the R 3.6.1 software was used to calculate the cumulative probability of the ranking results of all treatments, which can be seen in Figure 4. Node splitting analysis was used to test the consistency of some comparison results. According to the node splitting analysis in Supplementary File S9, all the *p*-values were greater than 0.05. This indicated that there was no statistical difference and that the models had good consistency.

### 3.5. Secondary outcomes

#### 3.5.1. Time to diarrheal disappearance

**Figure 5:**
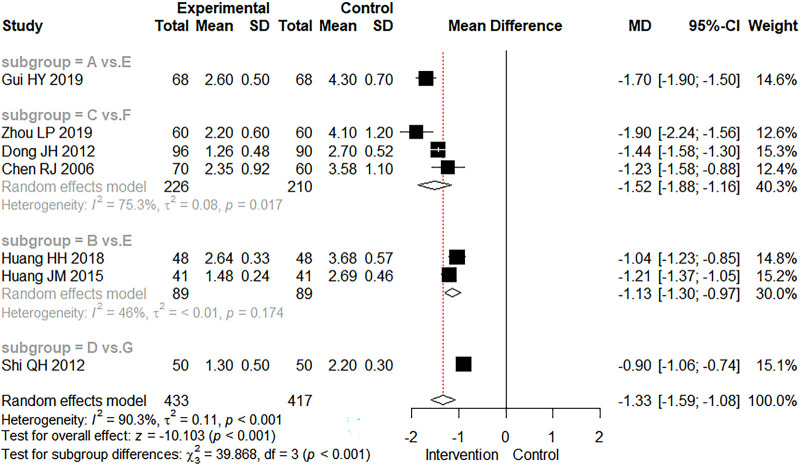
The forest plot of time to diarrheal disappearance. (**A**) Chinese medicine belly button application. (**B**) Chinese medicine belly button application plus montmorillonite powder. (**C**) Chinese medicine belly button application plus montmorillonite powder plus microecologics. (**D**) Chinese medicine belly button application plus montmorillonite powder plus anti-infectives. (**E**) montmorillonite powder. (**F**) montmorillonite powder plus microecologics. (**G**) montmorillonite powder plus anti-infectives.

A total of seven studies reported the time to diarrhea disappearance. Because the treatment measures in these seven studies could not form a complete network, a direct meta-analysis was used. The meta-analysis results are shown in Figure 5, along with the combined test analysis results (MD = −1.33 days, 95% CI: −1.59 to −1.08, *Z* = −10.103, *p* < 0.001). These results indicated that either the exclusive use of CMBBA or modern medicine in combination with CMBBA were effective in reducing the time to diarrheal disappearance compared to modern medicine, and the difference was statistically significant. As can be seen from Figure 5, studies involving the time to diarrhea disappearance were generally heterogeneous (*I*^2^ = 90.3%, *p* < 0.001), so we also performed subgroup analysis based on treatment measures in Figure 5. Heterogeneity was significantly reduced after subgroup analysis. The results suggested that treatment measures may be the main source of heterogeneity in the time to diarrheal disappearance. Sensitivity analysis was also performed for the time to diarrhea disappearance, and the results are shown in Supplementary File S5, showing that the sensitivity of all studies was relatively robust and reliable.

#### 3.5.2. Recovery time of dehydration

**Figure 6:**
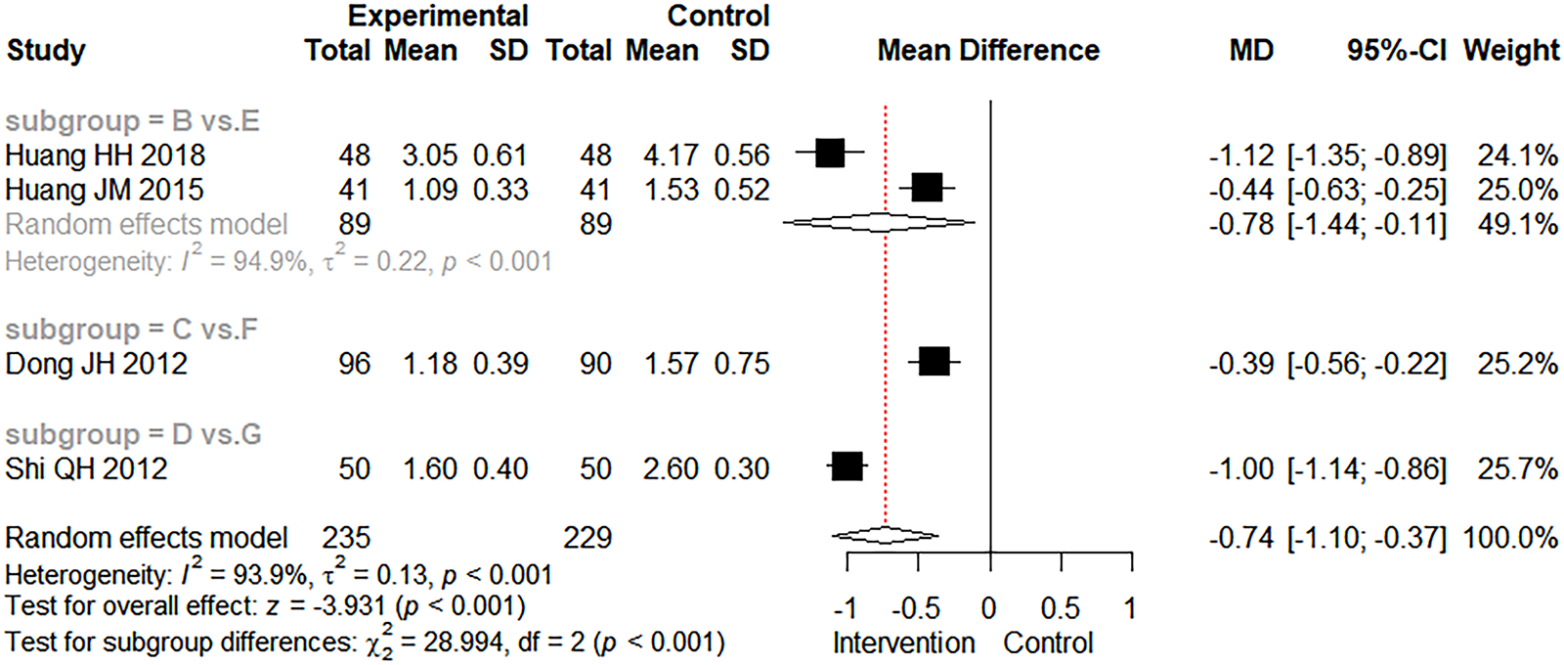
The forest plot of the recovery time of dehydration. Group (**B**) Chinese medicine belly button application plus montmorillonite powder. Group (**C**) Chinese medicine belly button application plus montmorillonite powder plus microecologics. Group (**D**) Chinese medicine belly button application plus montmorillonite powder plus anti-infectives. Group (**E**) Montmorillonite powder. Group (**F**) Montmorillonite powder plus microecologics. Group (**G**) Montmorillonite powder plus anti-infectives.

There were four studies that mentioned the recovery time of dehydration. Figure 6 depicts the results of direct meta-analysis, as were the results of comprehensive analysis (MD = −0.74 days, 95% CI: −1.10 to −0.37, *Z* = −3.931.103, *p* < 0.001). It showed that the combined use of CMBBA could reduce the recovery time of dehydration by an average of 0.74 days compared to modern medicine. As can be seen in Figure 6, the heterogeneity was relatively high for the two studies involving CMBBA plus montmorillonite powder vs. montmorillonite powder. After comparing the two studies, we found that one study used CMBBA twice a day, and the other study used CMBBA once a day. The other two studies were similar in terms of treatment and baseline. We speculated that the frequency of using CMBBA may be a source of heterogeneity between the two studies. We also conducted a sensitivity analysis on the recovery time for dehydration, as shown in Supplementary File S5. This was still statistically significant after one-by-one omissions from the study, indicating that the results were relatively robust and reliable.

#### 3.5.3. Adverse events

**Figure 7:**
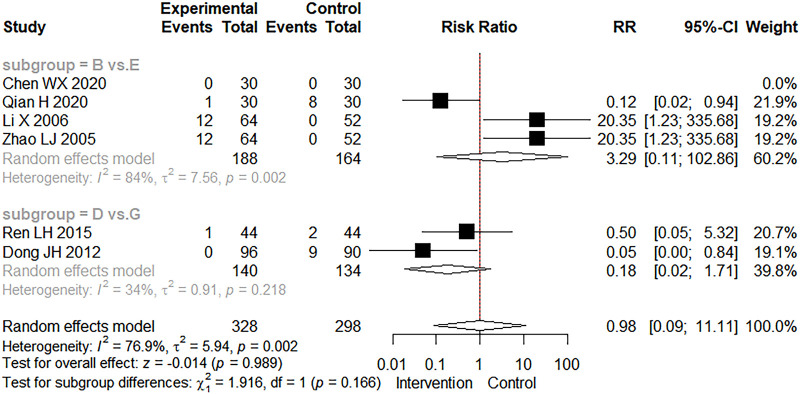
The forest plot of adverse events. Group (**B**) Chinese medicine belly button application plus montmorillonite powder. Group (**D**) Chinese medicine belly button application plus montmorillonite powder plus anti-infectives. Group (**E**) Montmorillonite powder. Group (**G**) Montmorillonite powder plus anti-infectives.

The occurrence of adverse events was mentioned in six studies. In Figure 7, we found no statistically significant difference in adverse events between the intervention and control groups (RR = 0.98, 95% CI: 0.09–11.11, *Z* = −0.014, *p* = 0.989). Adverse events have been reported with the use of CMBBA, including mild allergic reactions and mild abdominal pain, but they can heal on their own in a relatively short period of time. Therefore, the use of CMBBA was relatively safe.

### 3.6. Publication bias analysis

**Figure 8:**
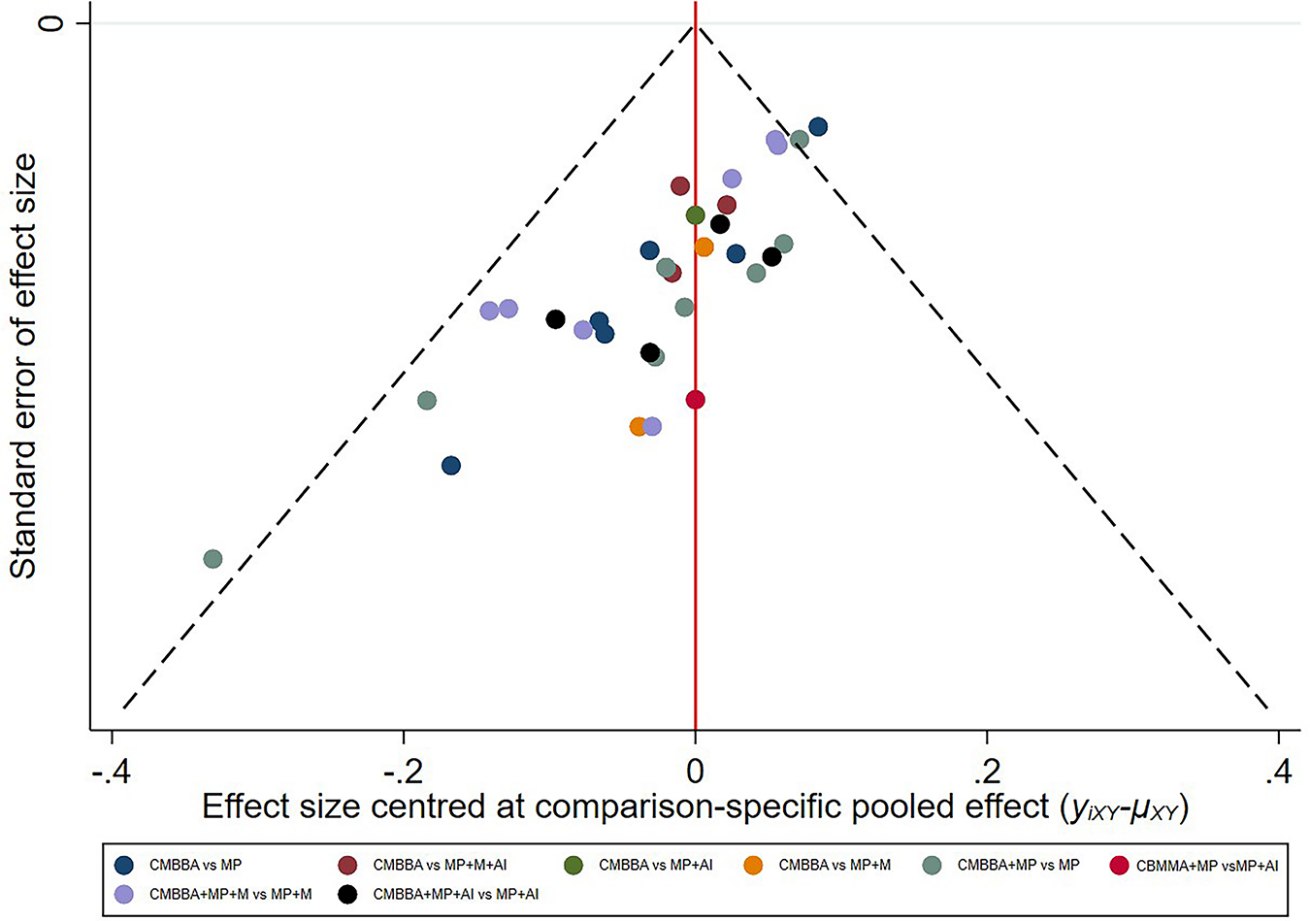
The publication bias plots for clinical effectiveness. CMBBA, Chinese medicine belly button application; MP, montmorillonite powder; M, microecologics; AI, anti-infectives.

The publication bias chart for clinical effectiveness can be seen in Figure 8, and the publication bias chart for other outcome measures can be seen in Supplementary File S10. We found that the distribution of scatter points in the funnel plot of all outcome measures was symmetric and that the number of scatter points on the left and right sides was equal. We used the Peters test to test the publication bias of clinical effectiveness and Egger linear regression to test the publication bias of other outcome measures, and the results showed that all outcome measures had no publication bias (clinical effectiveness: *p* = 0.1835; time to diarrheal disappearance: *p* = 0.4501; recovery time of dehydration: *p* = 0.8654; adverse event: *p* = 0.3348).

### 3.7. Confidence in evidence

We used CINeMA to evaluate the clinical effectiveness of each treatment. The results can be seen in Supplementary CINeMA results. According to the evaluation results, the evaluation results were “moderate,” “low,” and “very low,” with “very low” appearing relatively more. We also used contribution plots to identify the direct comparisons that most influence the NMA merger results. We found that the evaluation results among the other treatment measures were “moderate” except for the very low evaluation results between CMBBA and montmorillonite powder plus anti-infectives and the “low” evaluation results between CMBBA and montmorillonite powder plus anti-infectives plus microecologics. Combined with the contribution figure in Supplementary File S11, the direct comparison of CMBBA and montmorillonite powder accounted for 18.5% of the entire network. Therefore, the results between CMBBA and montmorillonite powder should be treated with caution.

### 3.8. Results of a minimally contextualised framework

Initially, we chosen montmorillonite powder plus microecologics as the reference group since high or moderate certainty evidence was more conducive to discerning treatment effects compared to evidence with low or very low certainty. Subsequently, we categorized the treatments into two groups based on whether they were statistically significant compared to the reference group. Group 0 included montmorillonite powder, montmorillonite powder plus anti-infectives, and montmorillonite powder plus anti-infectives plus microecologics, which was not statistically significant compared to the reference group. In contrast, Group 1 comprised treatments that demonstrated statistical significance, specifically CMBBA, CMBBA plus montmorillonite powder, CMBBA plus montmorillonite powder plus microecologics, and CMBBA plus montmorillonite powder plus anti-infectives. Then, we categorized the treatments into two categories: evidence with high/moderate certainty and evidence with low certainty. Finally, we included SUCRA values for each group. Table 3 shows the results of the specific steps. From Table 3, we can conclude that compared to montmorillonite powder plus microecologics, CMBBA, CMBBA plus montmorillonite powder, CMBBA plus montmorillonite powder plus microecologics, and CMBBA plus montmorillonite powder plus anti-infectives are all effective treatment options.

**Table 3:** Final classification of treatments, based on network meta-analysis of treatments for childhood diarrhea.

Certainty of the evidence, and classification of intervention^a^	Category	Treatments	Treatments vs. the reference treatments (Relative risk/95% credible interval)	Surface under the cumulative ranking curve
High certainty (moderate to high certainty evidence)	Category 1	CMBBA	1.14 (1.06, 1.23)	0.68
		CMBBA + MP	1.2 (1.08, 1.33)	0.86
		CMBBA + MP	1.13 (1.08, 1.21)	0.68
		CMBBA + MP + M	1.13 (1.08, 1.21)	0.68
		CMBBA + MP + AI	1.23 (1.01, 1.48)	0.88
	Category 0	–		
Low certainty (low to very low certainty evidence)	Category 1	–		
	Category 0	MP	1.02 (0.93, 1.12)	0.32
		MP + AI	1.02 (0.85, 1.2)	0.31
		MP + M + AI	0.9 (0.8, 1.02)	0.02

^a^ Certainty of the evidence was determined based on Supplementary CINeMA results. CMBBA, Chinese medicine belly button application; MP, montmorillonite powder; M, microecologics; AI, anti-infectives.

## 4. Discussion

It’s documented that CMBBA is simple, convenient, effective, inexpensive, and easy for children to accept (53). In order to find more evidence-based medical evidence on the treatment of CD with CMBBA, according to the RCTs published in recent years on treating CD with CMBBA, we conducted the first NMA to evaluate the safety and effectiveness of CMBBA. This study analyzed data from 33 studies published between 1999 and 2020. This included 4,490, children with diarrhea: 2,319 in the control group and 2,171 in the intervention group.

In terms of clinical effectiveness, the clinical effectiveness of using CMBBA exclusively or CMBBA in combination with modern medicine to treat CD was found to be strikingly superior to using modern medicine exclusively. Combined with the SUCRA value, CMBBA plus montmorillonite powder plus anti-infectives may provide superior clinical effectiveness for children with diarrhea and concurrent infection. Additionally, a minimally contextualized framework showed that CMBBA plus montmorillonite powder plus anti-infectives also performed better among various treatments. From the studies included in the NMA, we found that the anti-infectives included specific drugs such as ribavirin, Moroxydine, acyclovir, and antibiotics. As an efficient protective agent of the digestive tract mucosa, montmorillonite powder has been shown to have significant clinical effects on layered structure and inhomogeneous charge distribution, as well as fibrinogen activation and increased antibacterial ability in children. At the same time, it is also able to suppress the phenomenon of pathogenic microorganisms damaging the epithelium of the gut, so that a corresponding therapeutic effect can be quickly achieved, thus reducing the frequency of stools and balancing the effect of water in the body of the child (54). Anti-infectives also play an important role in the treatment of CD, including antiviral and antibacterial infections. For CD, it is necessary to first distinguish whether they are infected diarrhea based on routine stool and blood examination, and further distinguish whether they are viral or bacterial infections after confirming infectious diarrhea, and select drugs according to different types. So far, there have been several studies that prove that the combination of anti-infectives and montmorillonite powder can effectively improve the clinical effectiveness of CD and significantly shorten the time of fever reduction, diarrheal disappearance, and stool recovery to normal (55, 56). Combining CMBBA with montmorillonite powder and anti-infectives may have a positive impact on multiple aspects, improve clinical effectiveness and shorten the duration of disease. Although this combination has shown the best clinical results in all treatment options, while it is not clinically appropriate for all children with diarrhea. Children with acute infectious diarrhea, the watery stool, an obvious toxic symptom and cannot completely explain through dehydration should be clinically treated with anti-infectives, especially in young infants and those with low immune function. Children with mucopurulent and bloody stools are mostly infected with invasive bacteria, which should be given antibiotics (57). Therefore, during clinical treatment, doctors should choose an appropriate treatment plan in the light of the specific condition of the child.

According to our study data, the second-best clinical effectiveness was CMBBA plus montmorillonite powder (SUCRA: 86%). A minimally contextualized framework demonstrated that CMBBA plus montmorillonite powder is also an effective treatment for CD. Gong (58) found that the use of CMBBA plus montmorillonite powder in the treatment of CD can improve the clinical effectiveness, the daily frequency of stool is significantly reduced, and the clinical symptoms such as vomiting and abdominal pain are significantly improved. The mechanism of microecologics preparation is to promote the reproduction and growth of normal dominant bacteria through beneficial bacteria and at the same time antagonize the proliferation of pathogenic bacteria so as to achieve the purpose of adjusting intestinal flora, maintaining the relative balance of microecology, preventing diseases, and improving health level (59).

Although our study found that the SUCRA value of CMBBA plus montmorillonite powder plus microecologics (SUCRA: 68%) was lower than that of CMBBA plus montmorillonite powder (SUCRA: 86%) and that of montmorillonite powder plus microecologics (SUCRA: 24%) than that of montmorillonite powder exclusively (SUCRA: 32%), from Table 2, there was no statistically significant difference between CMBBA plus montmorillonite powder plus microecologics and CMBBA plus montmorillonite powder (RR = 0.95, 95% CI: 0.88–1.07). Moreover, there was no statistically significant difference between montmorillonite powder plus microecologics and montmorillonite powder exclusively (RR = 0.98, 95% CI: 0.89–1.08). Furthermore, it is possible that due to the short course of treatment in the study, the balancing effect of probiotics on gut microecology did not show up in the short term, which led to the results. Wang et al. (60) found that the combination of microecologics and montmorillonite powder in the treatment of CD can play a synergistic role, strengthening the protective effect of the intestinal mucosa, further promoting the stability of the intestinal environment, reducing oxidative stress damage, and effectively improving clinical effectiveness. Therefore, combining the advantages of microecologics, we still recommend CBMMA combined with montmorillonite powder and microecologics to treat CD.

In addition, we also found that montmorillonite powder plus microecologics plus anti-infectives had the lowest clinical effectiveness (SUCRA: 0.02%) in the treatment of CD, because most anti-infectives in clinical practice have broad spectrum bactericidal effect, killing pathogens at the same time may harm beneficial bacteria, resulting in a decline in the diversity of intestinal flora, affecting the function of intestinal flora (61), so the combination is not very effective.

Overall, the heterogeneity of clinical effectiveness was low. We also performed subgroup analysis and sensitivity analysis and found that differences in treatment methods, dehydration, and treatment course may be the main sources of heterogeneity in clinical effectiveness. In terms of secondary outcomes, we found that compared with modern medicine, CMBBA exclusively or CMBBA combined with modern medicine could shorten the time to diarrheal disappearance of CD (MD = −1.33 days, 95% CI: −1.59 to −1.08, *Z* = −10.103, *p* < 0.001) and shortened the recovery time of dehydration (MD = −0.74 days, 95% CI: −1.10 to −0.37, *Z* = −3.931.103, *p* < 0.001), and the differences were statistically significant. Some studies have also reported adverse events associated with the use of CMBBA, such as mild rash or abdominal pain. However, the difference was not statistically significant compared with the control group (RR = 0.98, 95% CI: 0.09–11.11, *Z* = −0.014, *p* = 0.989), and these adverse reactions were self-healing and returned to normal within a short time. After subgroup analysis of secondary outcomes, we found that treatment style and frequency of treatment were the main sources of heterogeneity. The sensitivity analysis results showed that our results were robust and reliable.

This study has some strengths. Firstly, RCTs, as the highest level of evidence in evidence-based medicine, have been regarded as the “gold standard” in clinical evidence. We included only RCTs in this NMA, which improved confidence in the NMA results. Secondly, the two authors extracted, evaluated, and analyzed the data separately. If there were different opinions, the third author would be invited to discuss, solve, and finally reach an agreement, avoiding bias as much as possible. For the data analysis, we used CINeMA to assess the credibility of the evidence. Subgroup analysis and sensitivity analysis were performed to find sources of heterogeneity. A combination of pairwise meta-analysis and NMA was used. ROB 2.0 was used for methodological evaluation. Funnel plots were used to detect publication bias in outcome measures. We employed a minimally contextualized framework to provide a comprehensive conclusion for the NMA. But we also found some problems. For example, ROB 2.0 bias analysis results show that most studies have “some concerns”. The evidence grade evaluation result of the CINeMA part was low, and the heterogeneity of some outcome measures was high. Most studies involved fewer outcome measures, and the included studies lack foreign studies. In the future, we hope that more high-quality RCTs of CMBBA will be produced for CD, both domestically and internationally, to make up for our NMA shortcomings.

## 5. Conclusions

According to our findings, using CMBBA exclusively or combined with modern medicine have a positive effect on the treatment of CD. Based on minimally contextualized framework, the combination of CMBBA, montmorillonite powder, and anti-infectives may provide superior clinical effectiveness for children with diarrhea and concurrent infection. In addition, CMBBA reduced the time to diarrheal disappearance and the recovery time of dehydration in CD. While some studies reported adverse events to the use of CMBBA, the adverse events were mild and self-healing, and there was no statistical difference between the control and intervention groups. In summary, CMBBA is effective and safe for treating CD. Therefore, the authors recommend that the treatment of CD can be combined with the use of CMBBA, which would be beneficial for children with diarrhoea. However, due to the small number of clinical trials included and the low quality of these studies, future high-quality RCTs are critical to validating the effectiveness and safety of CMBBA for treating CD. In addition, due to the different conditions and causes of illness among individual patients, the choice of treatment plan should also be based on the specific conditions of each patient.

## Data Availability

The original contributions presented in the study are included in the article/**Supplementary Material**, further inquiries can be directed to the corresponding authors.
