# Supplementary material for: Comparative effectiveness and safety of Chinese medicine belly button application for childhood diarrhea: a Bayesian network meta-analysis of randomized controlled trials
Source: Front Pediatr. 2023 Aug 4;11:1180694. doi: 10.3389/fped.2023.1180694 (PMC10436529; doi:10.3389/fped.2023.1180694)
Supplement: Supplementary file 2 [file Datasheet2.pdf]

Supplementary Material

Comparative effectiveness and safety of Chinese medicine belly button application for childhood diarrhea: A bayesian network meta-analysis of randomized controlled trials

Zhi-jun Bu[1†], Yan-ni Liu[2†], Md. Shahjalal[3], You-you Zheng[1], Cheng-jiang Liu[4], Meng-meng Ye[5], Jin-yang Xu[1], Xin-yao Peng[1], Xue-hui Wang[1], Xu Chen[2], Jian-ping Liu[1], Hui-lan Liu[2*], and Zhao-lan Liu[1*]

†These authors contributed equally to this work and share first authorship.

*** Correspondence:** Prof. Huilan-Liu and Prof. Zhaolan-Liu, Centre for Evidence-Based Chinese Medicine, Beijing University of Chinese Medicine, Sunshine South Street, Fangshan District, Beijing 102400, P. R. China. Tel: (+86) 010-64286757,E-mail: lzl1019@163.com.

1 Supplementary file 1

Table file 1, PRISMA checklist for comparative effectiveness and safety of Chinese medicine belly button application for childhood diarrhea.

**Section/Topic**	**Item #**	**Checklist Item**	**Reported on Page #**
**TITLE**<br>Title	1	Identify the report as a systematic review incorporating a network meta-analysis (or related form of meta-analysis).	Page 1.
**ABSTRACT**<br>Structured summary	2	Provide a structured summary including, as applicable:<br>**Background:** main objectives<br>**Methods:** data sources; study eligibility criteria, participants, and interventions; study appraisal; and synthesis methods, such as network meta-analysis.<br>**Results:** number of studies and participants identified; summary estimates with corresponding confidence/credible intervals; treatment rankings may also be discussed. Authors may choose to	Page 1.

		summarize pairwise comparisons against a chosen treatment included in their analyses for brevity.<br><br>**Discussion/Conclusions:** limitations; conclusions and implications of findings.<br><br>**Other:** primary source of funding; systematic review registration number with registry name.	
**INTRODUCTION**<br><br>Rationale	3	Describe the rationale for the review in the context of what is already known, including mention of why a network meta- analysis has been conducted.	Page 2. In the **1. Introduction** section
Objectives	4	Provide an explicit statement of questions being addressed, with reference to participants, interventions, comparisons, outcomes, and study design (PICOS).	Page 3. In the **1. Introduction** section
**METHODS**<br><br>Protocol and registration	5	Indicate whether a review protocol exists and if and where it can be accessed (e.g., Web address); and, if available, provide registration information, including registration number.	Page 3. In the **2. Materials and methods** section
Information sources	6	Describe all information sources (e.g., databases with dates of coverage, contact with study authors to identify additional studies) in the search and date last searched.	Page 4. In the **2.2. Search strategy** of the **2. Materials and methods** section

Search	7	Present full electronic search strategy for at least one database, including any limits used, such that it could be repeated.	Page 4. In the 2.2. **Search strategy** of the **2. Materials and methods** section
Eligibility criteria	8	Specify study characteristics (e.g., PICOS, length of follow-up) and report characteristics (e.g., years considered, language, publication status) used as criteria for eligibility, giving rationale. Clearly describe eligible treatments included in the treatment network, and note whether any have been clustered or merged into the same node (with justification).	Page 3. In the 2.1. **Eligibility criteria** of the 2. **Materials and methods** section
Study selection	9	State the process for selecting studies (i.e., screening, eligibility, included in systematic review, and, if applicable,included in the meta-analysis).	Page 4. In the 2.3. **Study selection** of the **2.Materials and methods** section
Data collection process	10	Describe method of data extraction from reports (e.g., piloted forms, independently, in duplicate) and any processes for obtaining and confirming data from investigators.	Page 4. In the 2.3. **Study selection** of the **2.Materials and methods** section
Data items	11	List and define all variables for which data were sought (e.g.,PICOS, funding sources) and any assumptions and simplifications made.	Page 4. In the 2.3. **Study selection** of the **2.Materials and methods** section
**Geometry of the network**	**S1**	Describe methods used to explore the geometry of the treatment network under study and potential biases related to it.This should include how the evidence base has been graphically summarized for presentation, and what characteristics were compiled and used to describe the evidence base to readers.	Page 4. In the 2.5. **Data analysis** of the **2. Materials and methods** section

Risk of bias within individual studies	12	Describe methods used for assessing risk of bias of individual studies (including specification of whether this was done at the study or outcome level), and how this information is to be used in any data synthesis.	Page 4. In the 2.4. **Risk of bias assessment** of the **2. Materials and methods** section
Summary measures	13	State the principal summary measures (e.g., risk ratio, difference in means). Also describe the use of additional summary measures assessed, such as treatment rankings and surface under the cumulative ranking curve (SUCRA) values,as well as modified approaches used to present summary findings from meta-analyses.	Page 4. In the 2.5. **Data analysis** of the **2.Materials and methods** section
Planned methods of analysis	**14**	Describe the methods of handling data and combining results of studies for each network meta-analysis. This should include,but not be limited to:<br>Handling of multi-arm trials;<br>Selection of variance structure;<br>Selection of prior distributions in Bayesian analyses;<br>And Assessment of model fit.	Page 4. In the 2.5. **Data analysis** of the **2.Materials and methods** section
**Assessment of Inconsistency**	**S2**	Describe the statistical methods used to evaluate the agreement of direct and indirect evidence in the treatment network(s) studied. Describe efforts taken to address its presence when found.	Page 4. In the 2.5. **Data analysis** of the **2.Materials and methods** section
Risk of bias across studies	**15**	Specify any assessment of risk of bias that may affect the cumulative evidence (e.g., publication bias, selective reporting within studies).	Page 8. In the 3.6. **Analysis of publication bias** of the **3. Results** section

Additional analyses	**16**	Describe methods of additional analyses if done, indicating which were pre-specified. This may include, but not be limited to, the following: Sensitivity or subgroup analyses; Meta-regression analyses; Alternative formulations of the treatment network; and Use of alternative prior distributions for Bayesian analyses (if applicable).	Page 4. In the 2.5. **Data analysis** of the **2.Materials and methods** section
**RESULTS†**<br>Study selection	**17**	Give numbers of studies screened, assessed for eligibility, and included in the review, with reasons for exclusions at each stage, ideally with a flow diagram.	Page 5. In the 3.1. **Study selection** of the **3.Results** section
**Presentation of network structure**	**S3**	Provide a network graph of the included studies to enable visualization of the geometry of the treatment network.	Page 6. In the 3.4.2 **Network meta-analysis** of the **3.Results** section
**Summary of network geometry**	**S4**	Provide a brief overview of characteristics of the treatment network. This may include commentary on the abundance of trials and randomized patients for the different interventions and pairwise comparisons in the network, gaps of evidence in the treatment network, and potential biases reflected by the network structure.	Page 6. In the 3.4.2 **Network meta-analysis** of the **3.Results** section
Study characteristics	**18**	For each study, present characteristics for which data were extracted (e.g., study size, PICOS, follow-up period) and provide the citations.	Page 5. In the 3.2. **Study characteristics** of the **3.Results** section
Risk of bias within studies	**19**	Present data on risk of bias of each study and, if available, any outcome level assessment.	Page 5. In the 3.3. **Risk of bias of included studies** of the **3.Results** section

Results of individual studies	**20**	For all outcomes considered (benefits or harms), present, for each study: 1) simple summary data for each intervention group, and 2) effect estimates and confidence intervals. Modified approaches may be needed to deal with information from larger networks.	Page 5. In the **3.Results** section
Synthesis of results	**21**	Present results of each meta-analysis done, including confidence/credible intervals. In larger networks, authors may focus on comparisons versus a particular comparator (e.g. placebo or standard care), with full findings presented in an appendix. League tables and forest plots may be considered to summarize pairwise comparisons. If additional summary measures were explored (such as treatment rankings), these should also be presented.	Page 5. In the 3.4. **Clinical effectiveness and 3.5 Secondary outcomes** of the **3.Results** section
**Exploration for inconsistency**	**S5**	Describe results from investigations of inconsistency. This may include such information as measures of model fit to compare consistency and inconsistency models, P values from statistical tests, or summary of inconsistency estimates from different parts of the treatment network.	Page 6. In the 3.4.1 **Pairwise meta-analysis** of the **3.Results** section
Risk of bias across studies	**22**	Present results of any assessment of risk of bias across studies for the evidence base being studied.	Page 5. In the **3.3. Risk of bias of included studies** of the **3.Results** section
Results of additional analyses	**23**	Give results of additional analyses, if done (e.g., sensitivity or subgroup analyses, meta-regression analyses, alternative network geometries studied, alternative choice of prior distributions for Bayesian analyses, and so forth).	Page 5. In the 3.4. **Pairwise meta-analysis** of the **3.Results** section

**DISCUSSION**<br><br>Summary of evidence	**24**	Summarize the main findings, including the strength of evidence for each main outcome; consider their relevance to key groups (e.g., healthcare providers, users, and policy-makers).	Page 8. In the **4.Discussion** section
Strengths and limitation	**25**	Discuss limitations at study and outcome level (e.g., risk of bias), and at review level (e.g., incomplete retrieval of identified research, reporting bias). Comment on the validity of the assumptions, such as transitivity and consistency. Comment on any concerns regarding network geometry (e.g., avoidance of certain comparisons).	Page 10. In the **4.Discussion** section
Conclusions	**26**	Provide a general interpretation of the results in the context of other evidence, and implications for future research.	Page 10. In the **5.Conclusions** section
**FUNDING**<br><br>Funding	**27**	Describe sources of funding for the systematic review and other support (e.g., supply of data); role of funders for the systematic review. This should also include information regarding whether funding has been received from manufacturers of treatments in the network and/or whether some of the authors are content experts with professional conflicts of interest that could affect use of treatments in the network.	Page 11. In the **Funding** section

2 Supplementary file 2

Table file 2, The search strategy for the respective database.

Database name	Search strategies
CNKI	SU=('小儿腹泻'+'儿童腹泻'+'小儿泄泻'+'儿童泄泻'+'婴儿腹泻'+'婴儿泄泻'+'婴幼儿腹泻'+'婴幼儿泄泻') AND SU=('脐疗'+'中药敷贴'+'脐针'+'神阙敷贴'+'脐贴') AND FT='随机'
Wanfang	主题:("小儿腹泻" or "儿童腹泻" or "小儿泄泻" or "儿童泄泻" or"婴儿腹泻"or "婴儿泄泻") and 主题:("脐疗" or "中药敷贴"or "脐针" or "神阙敷贴" or "脐贴") and 全部:(随机)

VIP	(M=(小儿腹泻 or 儿童腹泻 or 小儿泄泻 or 儿童泄泻 or 婴儿腹泻 or 婴儿泄泻) or K=(小儿腹泻 or 小儿腹泻 or 小儿泄泻 or 儿童泄泻 or 婴儿腹泻 or 婴儿泄泻)) and (M=(脐疗 or 中药敷贴 or 脐针 or 神阙敷贴 or 脐贴) or K=(脐疗 or 中药敷贴 or 脐针 or 神阙敷贴 or 脐贴)) and U=随机
SinoMed	#1 “腹泻 婴儿”[不加权：扩展]<br>#2 “儿童”[不加权：扩展]<br>#3 (“腹泻”[不加权：扩展])(“泄泻”[不加权：扩展])<br>#4 (#3) AND (#2)<br>#5 (#4) OR (#1)<br>#6 “小儿腹泻”[常用字段：智能] OR “小儿泄泻”[常用字段：智能] OR “儿童腹泻”[常用字段：智能] OR “儿童泄泻”[常用字段：智能] OR “婴儿腹泻”[常用字段：智能] OR “婴儿泄泻”[常用字段：智能]<br>#7 (#6) OR (#5)<br>#8 “脐疗”[常用字段：智能] OR “中药敷贴”[常用字段：智能]OR “脐针”[常用字段：智能] OR “神阙敷贴”[常用字段：智能] OR “脐贴”[常用字段：智能]<br>#9 “随机”[全部字段：智能]<br>#10 (#9) AND (#8) AND (#7)
Pubmed	Search:(((“Diarrhea, Infantile”[Mesh]) OR ((childhood diarrhea) OR (infantile diarrhea))) AND (random*)) AND ((“Umbilicus”[Mesh]) OR (((((((Shen que) OR (navel) OR (umbilicus) OR (acupoint application)) OR (cupoint point application) OR (Chinese medicine external application)) OR (umbilical compress therapy)))
Cochrane Library	#1 Search:Mesh descriptor: [Diarrhea, Infantile] explode all trees<br>#2 Search: infantile diarrhea<br>#3 Search: childhood diarrhea<br>#4 Search: #1 OR #2 OR #3<br>#5 Search:Mesh descriptor: [Umbilicus] explode all tress<br>#6 Search:acupoint application<br>#7 Search:acupoint point application<br>#8 Search:Chinese medicine application<br>#9 Search:external application of chinese medicine<br>#10 Search:umbilical compress therapy<br>#11 Search:shenque<br>#12 Search:navel<br>#13 Search:umbilicus<br>#14 Search:#5 OR #6 OR #7 OR #8 OR #9 OR #10 OR #11 OR #12 OR #13<br>#15 Search:random<br>#16 Search:#4 AND #14 AND #15

Abbreviations in Table file 2, CNKI, China National Knowledge Infrast ructure; SinoMed, the Chinese Biomedical Literature Database; WanFang, the WanFang Database; VIP, the Chinese Scientific Journals Full-Text Database.

3 Supplementary file 3

The forest plot Pairwise Meta-Analysis of clincial effectiveness.

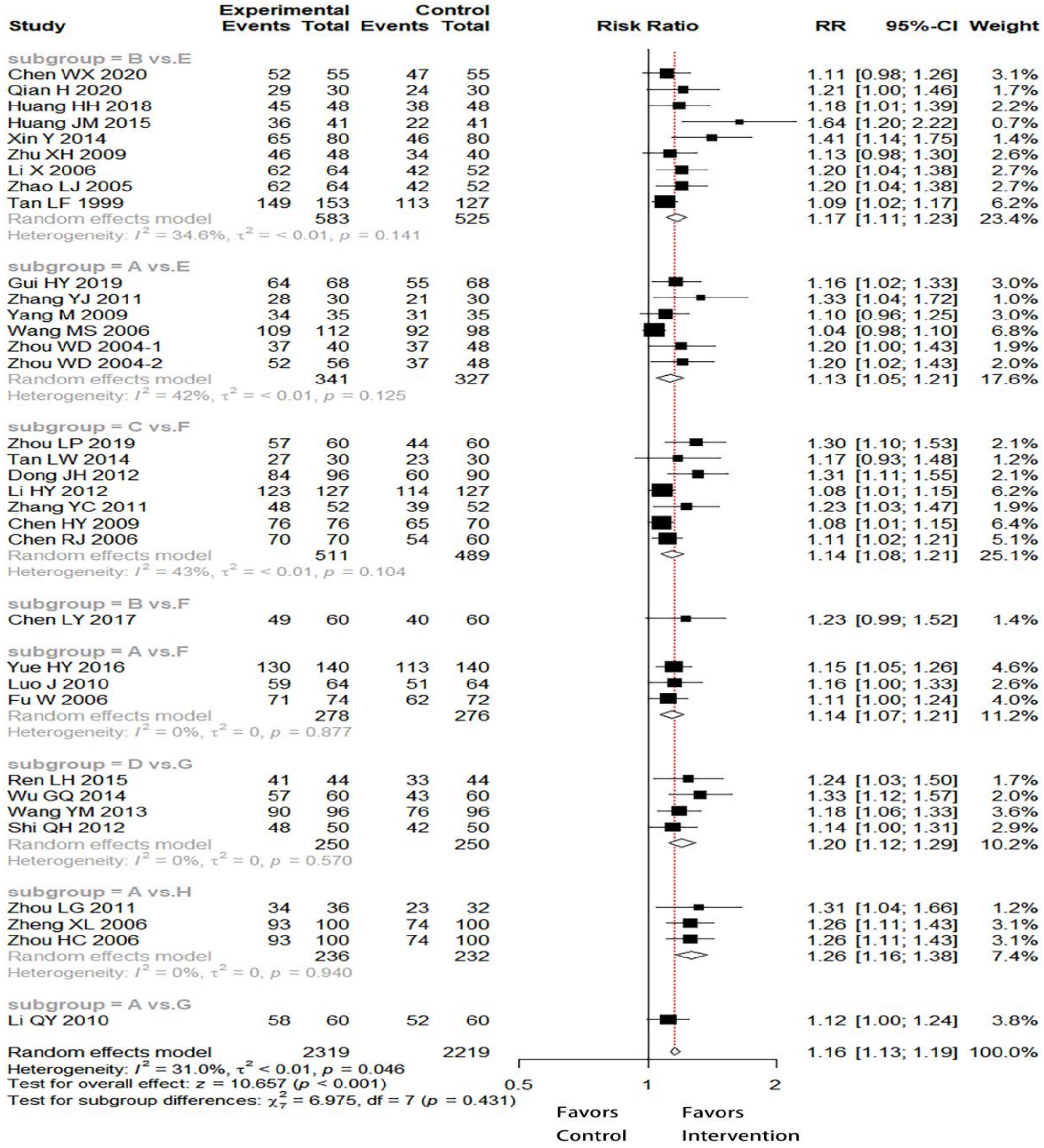

Supplementary Figure file 3, The forest plot Pairwise Meta-Analysis of clinical effectiveness. Abbreviations in Figure file 3, A: Chinese medicine belly button application. B: Chinese medicine belly button application plus montmorillonite powder. C: Chinese medicine belly button application plus montmorillonite powder plus microecologics. D： Chinese medicine belly button application plus montmorillonite powder plus anti-infectives. E： montmorillonite powder. F： montmorillonite powder plus microecologics. G： montmorillonite powder plus anti-infectives. H: montmorillonite powder plus anti-infectives plus microecologics.

4 Supplementary file 4

The subgroup analysis of clinical effectiveness.

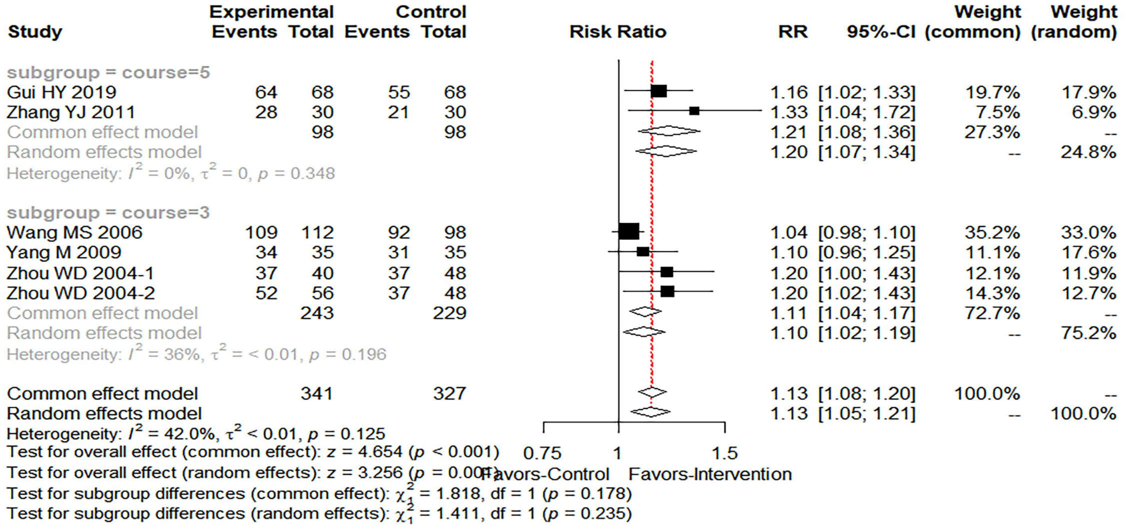

(A)

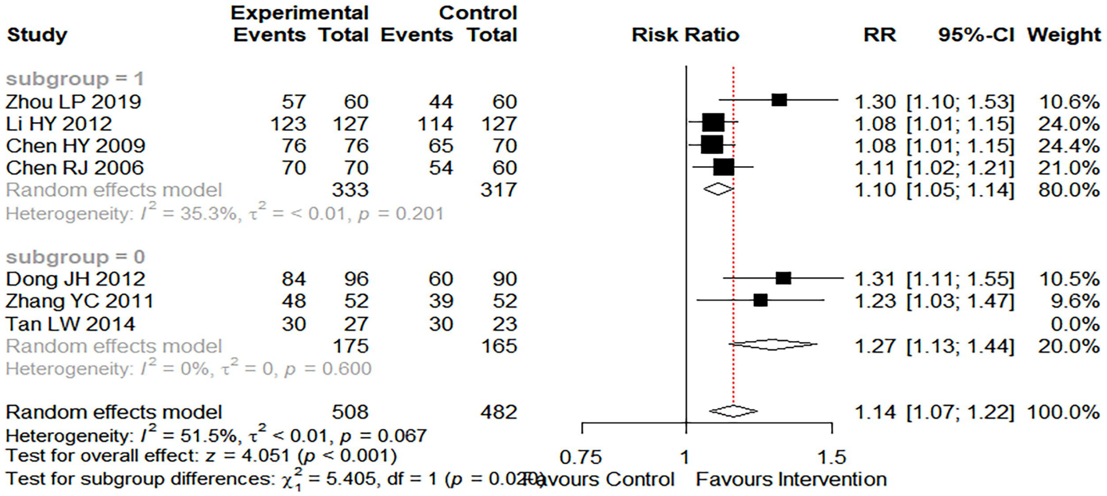

(B)

Supplementary Figure file 4, The subgroup analysis of clinical effectiveness. (A) Chinese medicine belly button application plus montmorillonite powder plus microecologics versus. montmorillonite powder. (B) Chinese medicine belly button application plus montmorillonite powder plus microecologics versus. montmorillonite powder plus microecologics.

5 Supplementary file 5

The sensitivity analysis results of each outcome meausre.

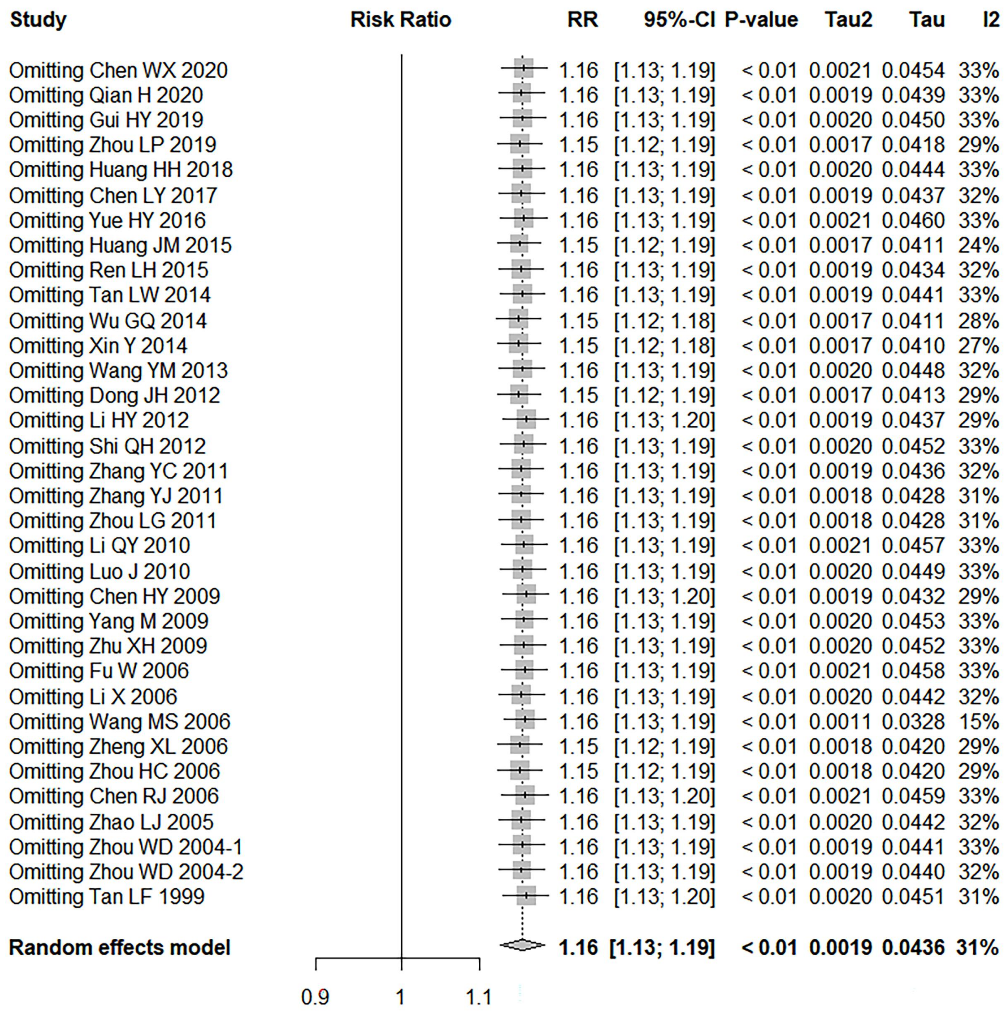

(A)

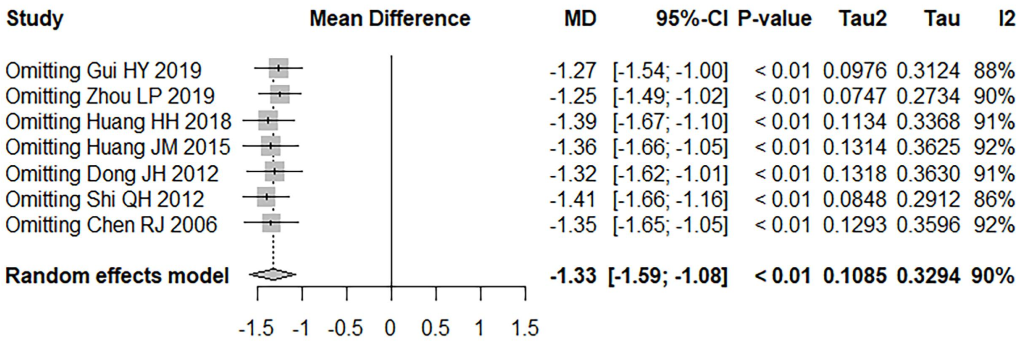

(B)

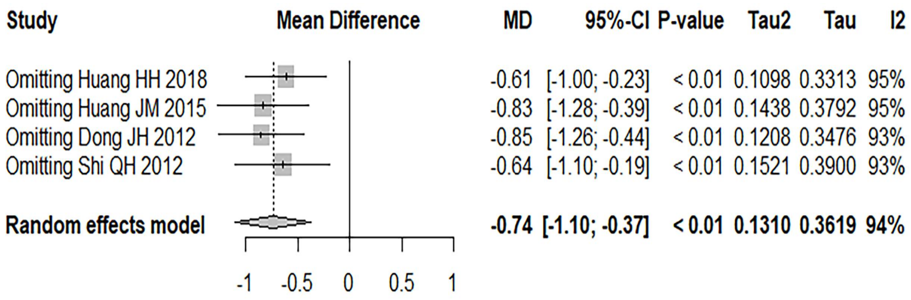

(C)

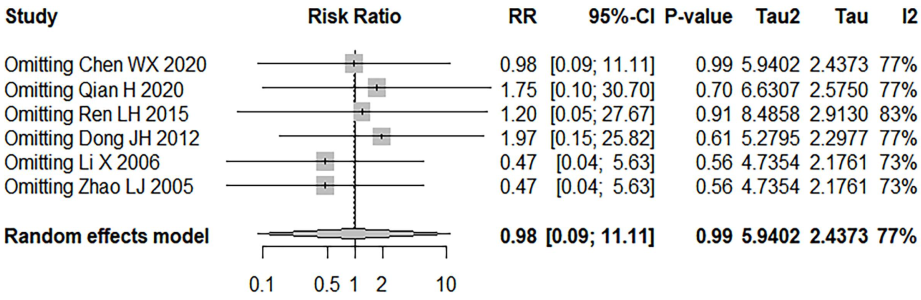

(D)

Supplementary Figure file 5, The sensitivity analysis results of each outcome meausre. (A) clinical effectiveness. (B) time to diarrheal disappearance. (C) recovery time of dehydration. (D) adverse events.

6 Supplementary file 6

The specific trajectory plots and density plots.

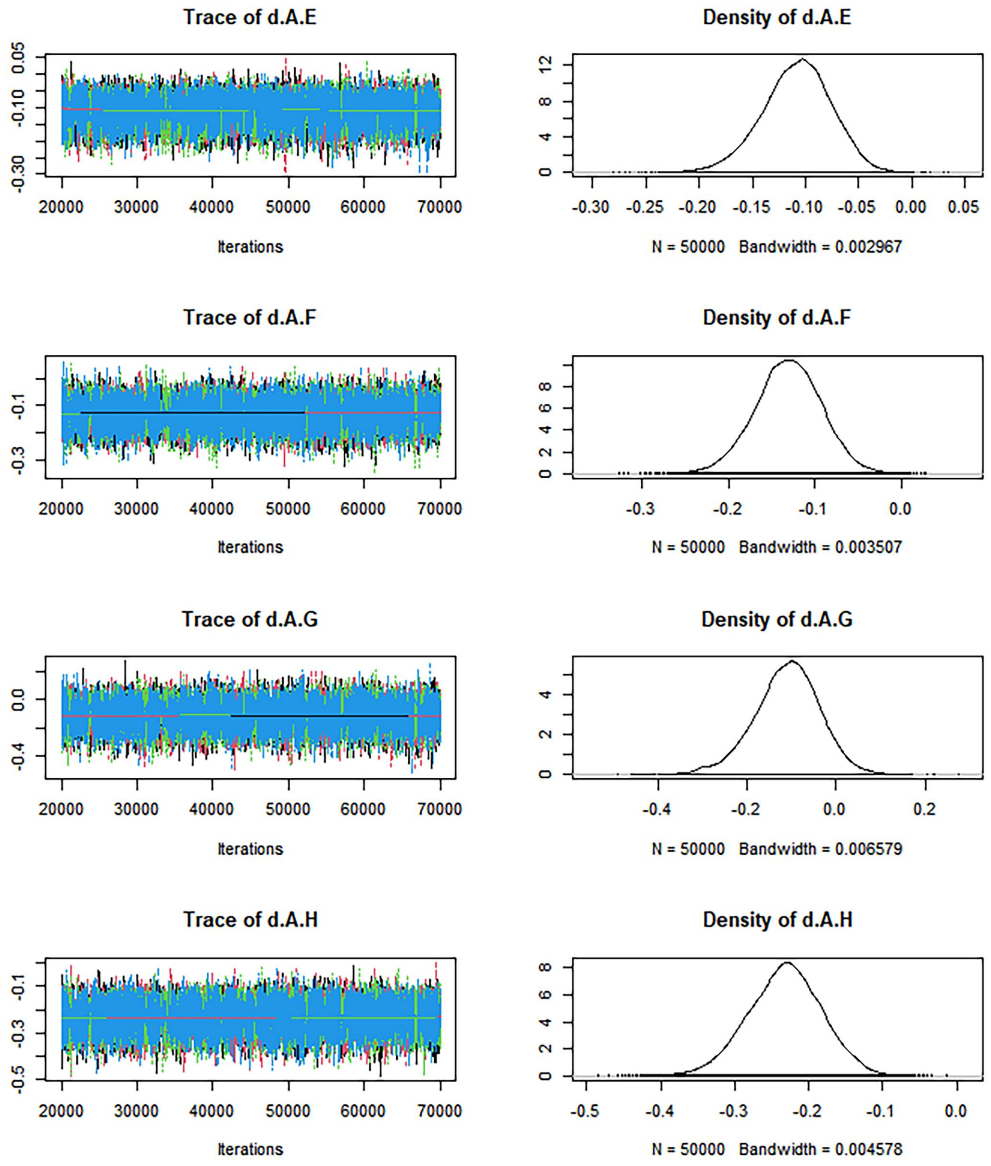

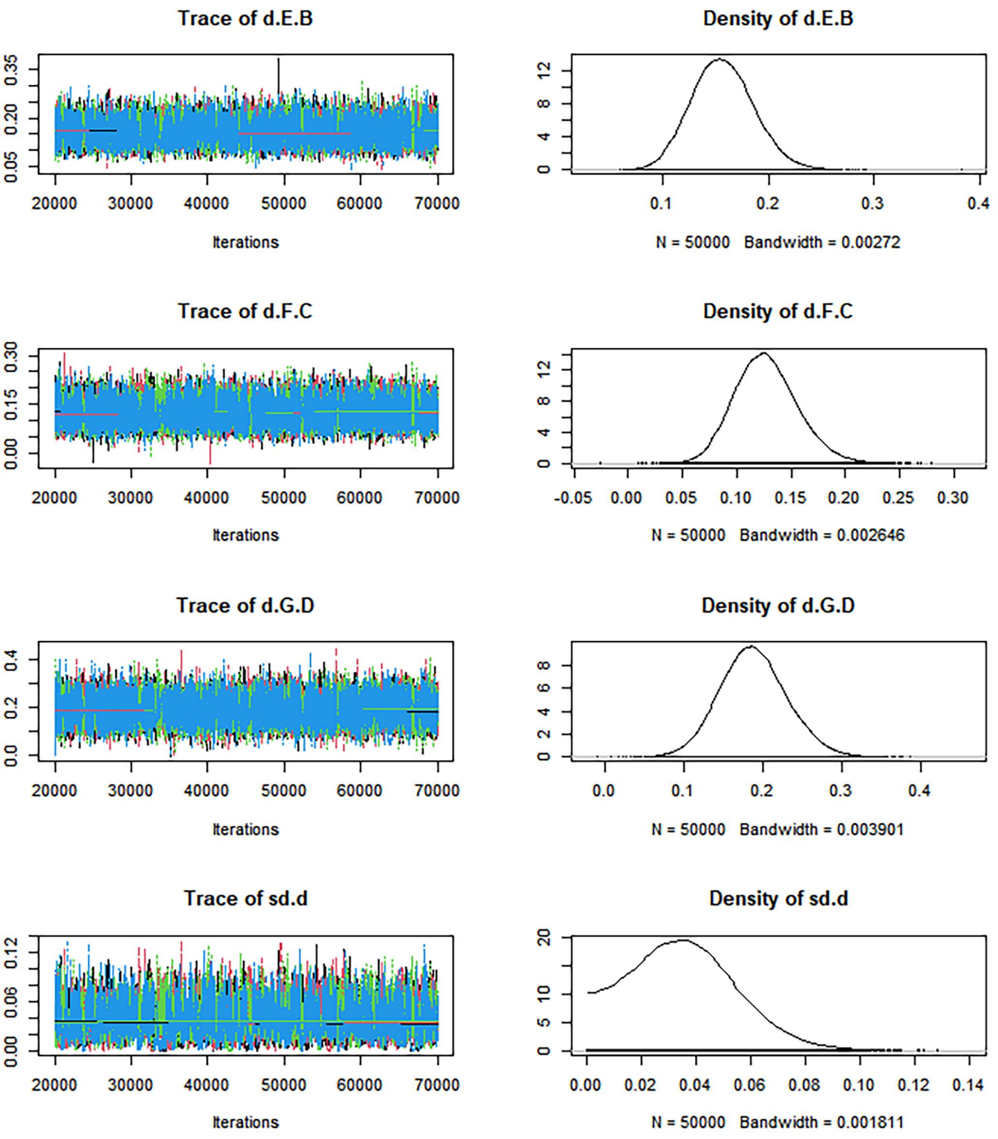

Supplementary Figure file 6, The specific trajectory maps and density plots of clincial effectiveness. Abbreviations in Figure file 6, A: Chinese medicine belly button application. B: Chinese medicine belly button application plus montmorillonite powder. C: Chinese medicine belly button application plus montmorillonite powder plus microecologics. D： Chinese medicine belly button application plus montmorillonite powder plus anti-infectives. E： montmorillonite powder. F： montmorillonite powder plus microecologics. G： montmorillonite powder plus anti-infectives. H: montmorillonite powder plus anti-infectives plus microecologics.

7 Supplementary file 7

Brooks-Gelman-Rubin diagnostic plots

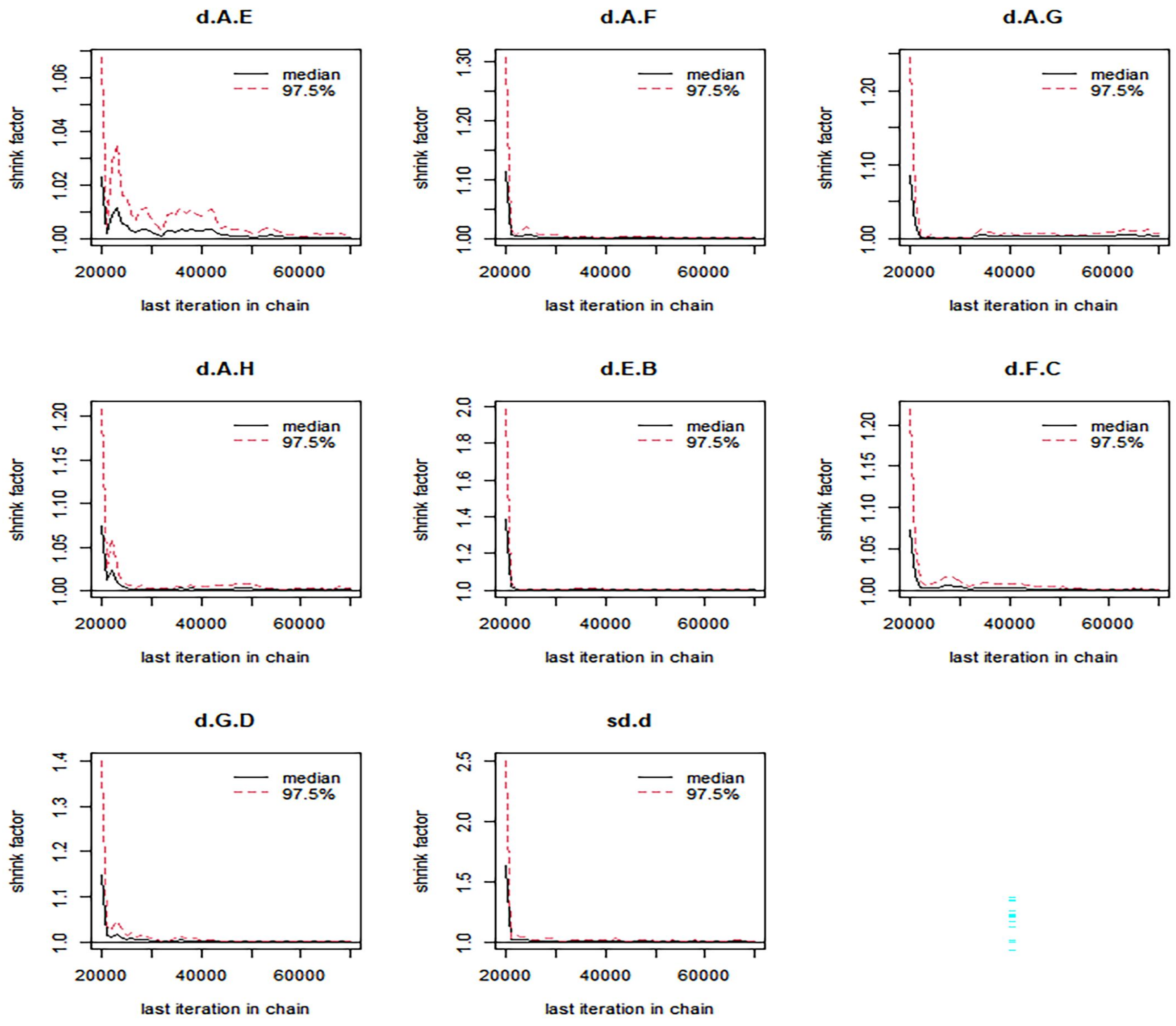

Supplementary Figure file 7, Brooks-Gelman-Rubin diagnostic plots of clincial effectiveness. Abbreviations in Figure file 7, A: Chinese medicine belly button application. B: Chinese medicine belly button application plus montmorillonite powder. C: Chinese medicine belly button application plus montmorillonite powder plus microecologics. D： Chinese medicine belly button application plus montmorillonite powder plus anti-infectives. E： montmorillonite powder. F： montmorillonite powder plus microecologics. G： montmorillonite powder plus anti-infectives. H: montmorillonite powder plus anti-infectives plus microecologics.

8 Supplementary file 8

The forest plot for network meta-analysis heterogeneity test.

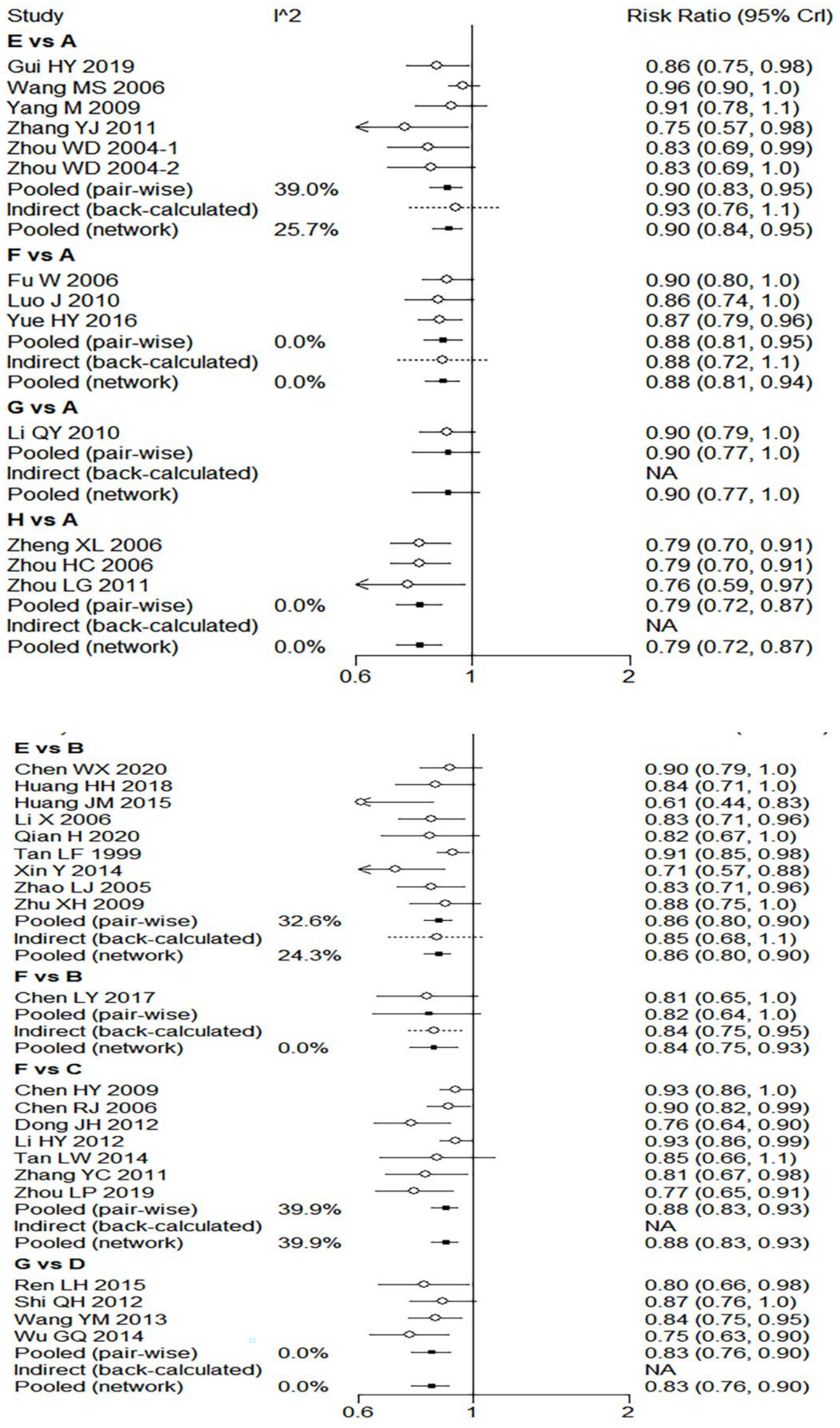

Supplementary Figure file 8, The forest plot for network meta-analysis heterogeneity test. Abbreviations in Figure file 8, A: Chinese medicine belly button application. B: Chinese medicine belly button application plus montmorillonite powder. C: Chinese medicine belly button application plus montmorillonite powder plus microecologics. D： Chinese medicine belly button application plus montmorillonite powder plus anti-infectives. E： montmorillonite powder. F： montmorillonite powder plus microecologics. G： montmorillonite powder plus anti-infectives. H: montmorillonite powder plus anti-infectives plus microecologics.

9 Supplementary file 9

The node splitting analysis of clinical effectiveness.

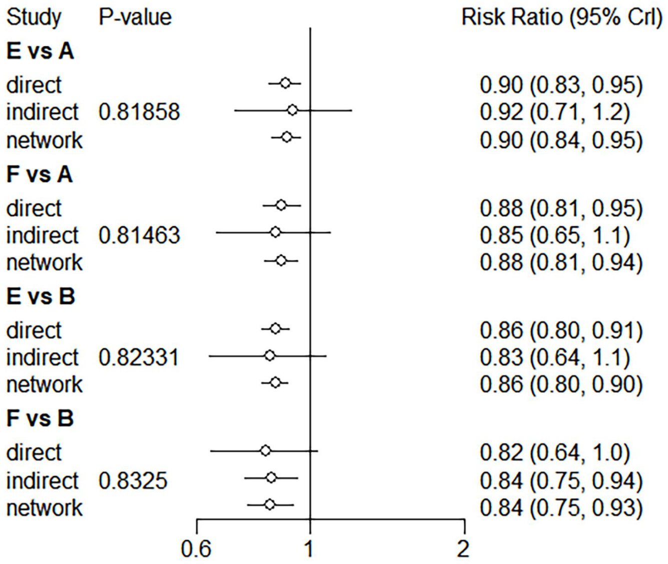

Supplementary Figure file 9, The node splitting analysis of clinical effectiveness. Abbreviations in Figure file 9, A: Chinese medicine belly button application. B: Chinese medicine belly button application plus montmorillonite powder. E： montmorillonite powder. F： montmorillonite powder plus microecologics.

10 Supplementary file 10

The publication bias chart for other outcome measures.

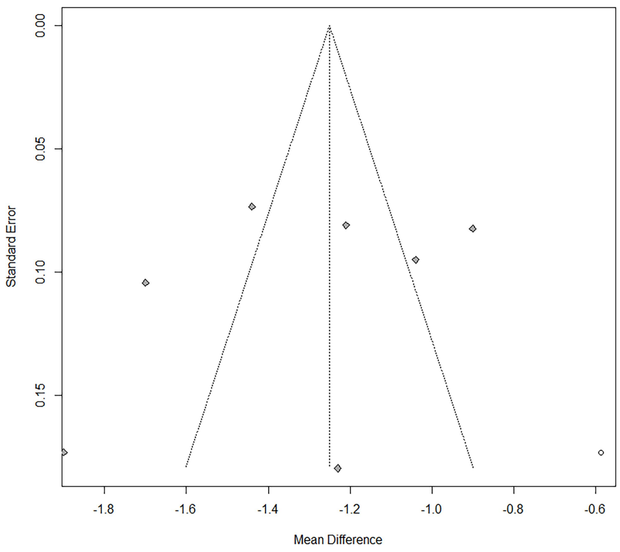

(A)

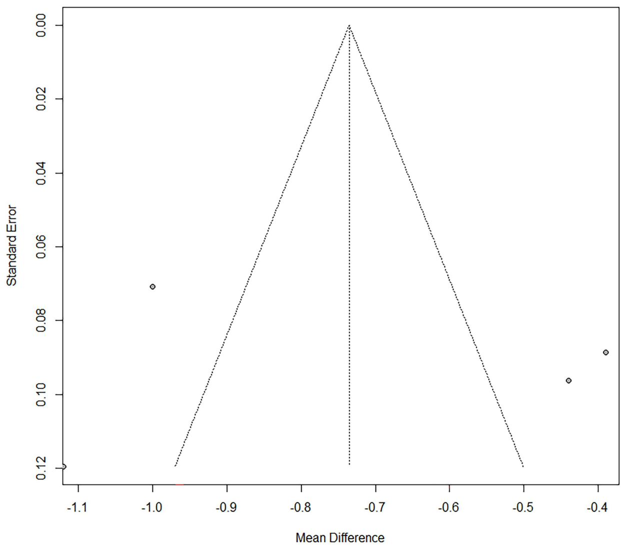

(B)

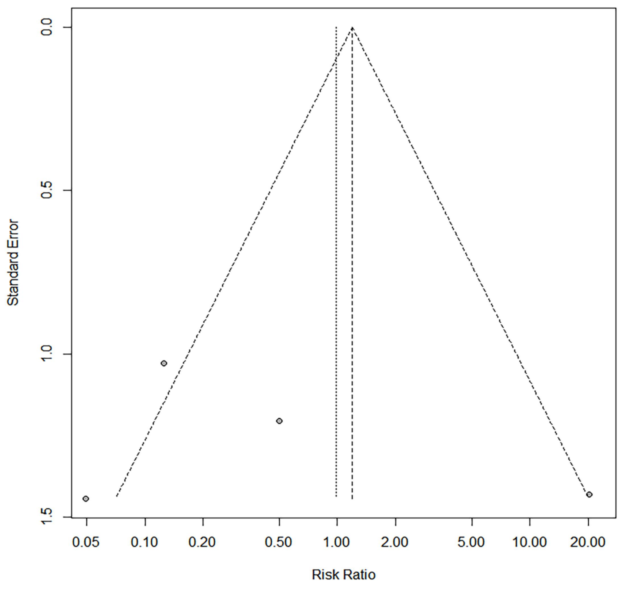

(C)

Supplementary Figure file 10, The publication bias chart for other outcome measures.(A) time to diarrheal disappearance. (B) recovery time of dehydration. (C) adverse events.

11 Supplementary file 11

Contribution plot for the clinical effectiveness.

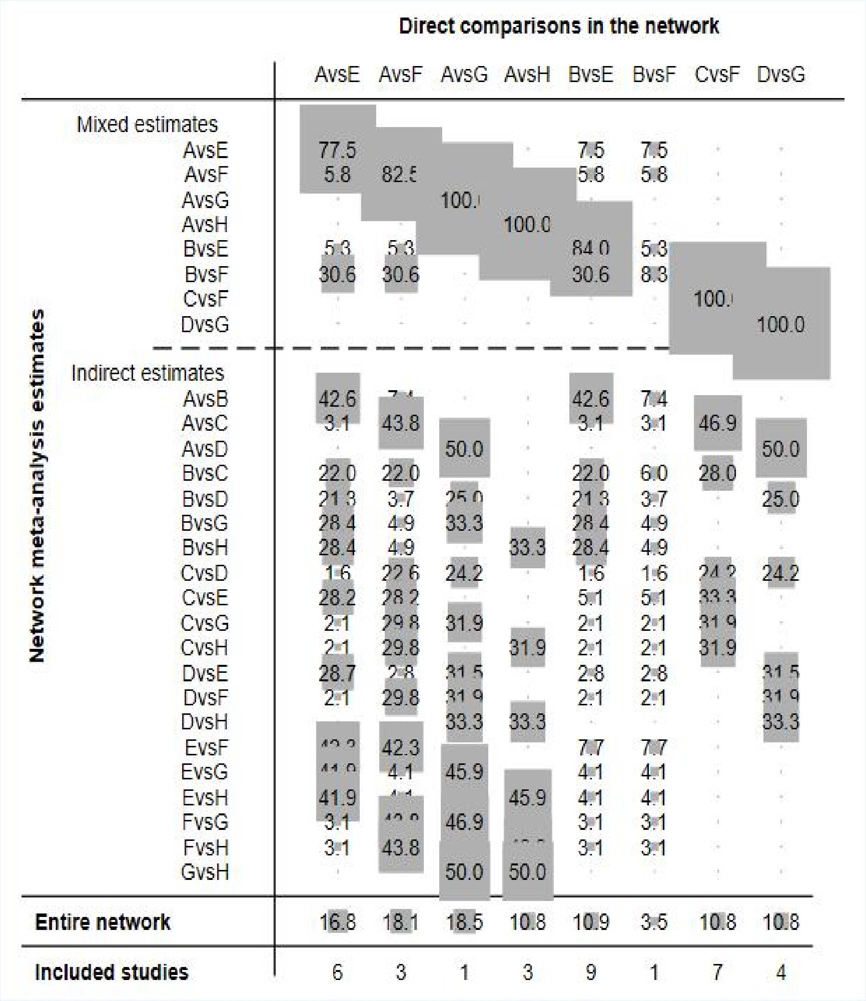

Supplementary Figure file 11, Contribution plot for the clinical effectiveness. The size of each square is proportional to the weight attached to each direct summary effect (horizontal axis) for the estimation of each network summary effects (vertical axis). The numbers re-express the weights as percentages. (A: Chinese medicine belly button application. B: Chinese medicine belly button application plus montmorillonite powder. C: Chinese medicine belly button application plus montmorillonite powder plus microecologics. D： Chinese medicine belly button application plus montmorillonite powder plus anti-infectives. E： montmorillonite powder. F： montmorillonite powder plus microecologics. G： montmorillonite powder plus anti-infectives. H: montmorillonite powder plus anti-infectives plus microecologics).

12 Supplementary file S12

The detailed information of 33 included studies in the network meta-analysis.

Number	Study Title	Author(s)	Year
1	Safety study of Chinese medicine preparation "Xiangren Navel Patch" in medical institutions	Chen WX	2020
2	Integrated Traditional Chinese and Western Medicine in Treating Infantile Diarrhea.	Qian H	2020
3	Clinical efficacy of Chinese herbal medicine self-formulated formula for umbilical cord application in the treatment of childhood diarrhea	Gui HY	2019
4	The efficacy of Chinese herbal compresses in synergy with Western medicine in the treatment of childhood autumn diarrhea.	Zhou LP, Yu CH, Xu XF, Xu GQ, Liu KW	2019
5	Clinical observation of 48 cases of childhood diarrhea treated with the aid of Chinese medicine applied to the umbilical cord.	Huang HH, Qin L, Dai YQ, Lin XX	2018
6	The efficacy of Wang's Bao Chi Pills applied to the umbilical cord in treating 60 cases of diarrhea in infants and children	Chen LY	2017
7	Effective observation on treating infantile diarrhea by umbilical compress based on TCM syndrome differentiation	Yue HY	2016
8	Clinical analysis of combined traditional Chinese and Western medicine in the treatment of childhood autumn diarrhea	Huang JM	2015
9	Efficacy of Chinese herbal medicine applied to the umbilical cord plus Simethicone enema in the treatment of childhood diarrhea.	Ren LH, Cui SZ	2015
10	Clinical efficacy of umbilical cord treatment for childhood diarrhea in 30 cases.	Tan LW	2014

11	Clinical effect analysis of topical umbilical paste in the treatment of pediatric diarrhea.	Wu GQ.	2014
12	Efficacy of umbilical cord therapy with Similac in the treatment of childhood prolonged diarrhea.	Xin Y, Suo YM	2014
13	Efficacy of Chinese herbal medicine applied to the umbilical cord as an aid in the treatment of childhood diarrhea.	Wang YM, He YX, Zhang YR, Liu JF	2013
14	Observation of 96 cases of autumn diarrhea in children treated with Chinese medicine and Western medicine.	Dong JH	2012
15	Umbilical Compress Therapy Combined with Smecta for 127 Cases of Acute Diarrhea in Infants	Li HY, Dong YN, Pan W.	2012
16	Experience of 100 cases of childhood diarrhea treated with a combination of Chinese medicine and Western medicine.	Shi QH	2012
17	Applying Granule of Chinese Medicine on Umbilicus to Treat ChiIdren Acute Diarrhea	Zhang YC	2011
18	Acupoint in treatment of acute diarrhea in children clinicalobservation of secondary lactose intolerance	Zhang YJ	2011
19	Study on the clinical efficacy of Chinese herbal medicine in the treatment of diarrheal diseases in infants and children.	Zhou LG	2011
20	60 cases of childhood autumn diarrhea treated with anti-diarrhea powder applied to the umbilical cord	Li QY	2010
21	Clinical and experimental study on the treatment of childhood diarrhea by applying childhood antidiarrheal powder to the umbilical cord.	Luo J, YU Y, Ran ZL	2010

22	Effectiveness of combined treatment of childhood diarrhea with Chinese herbal compresses on Shen Que point and foot San Li injection	Chen HY	2009
23	Treatment of 35 cases of childhood diarrhea with the combination of warming Chinese diarrhea relief powder and Yunnan Baiyao compresses.	Yan M	2009
24	48 cases of childhood diarrhea treated with childhood diarrhea with SiM.	Zhu XH	2009
25	Analysis of the efficacy of Chinese herbal medicine applied to the umbilical cord in the treatment of pediatric autumn diarrhea	Fu W, He ZB, Ke YB	2006
26	64 cases of diarrhea in infants and children treated with a combination of traditional Chinese and Western medicine	Li X, Wang GS, Zhang DQ.	2006
27	112 cases of childhood diarrhea treated with self-prepared anti-diarrheal spirit applied to the umbilical cord.	Wang MS	2006
28	The efficacy of Chinese herbal medicine applied to the umbilical cord in the treatment of pediatric diarrhea.	Zheng XL	2006
29	Clinical and experimental study on the treatment of childhooddiarrhea by applying anti-diarrheal compressing spirit to the umbilical cord	Zhou HC, Li HY, Chen HY, Zhao SZ	2006
30	Clinical efficacy of Chinese and western medicine applied to the umbilical cord for the treatment of autumn diarrhea in infants and young children.	Chen RJ, Yu Z, Wu WQ	2006
31	The efficacy of combined Chinese and Western medicine in treating 64 cases of diarrhea in infants and young children	Zhao LJ, Chen Y	2005

32	The efficacy of anti-diarrheal cream applied to the umbilical cord in treating 96 cases of autumn diarrhea in infants and children	Zhou WD	2004
33	153 cases of autumn diarrhea in infants treated with navel warming cream	Tan LF	1999
